# Meiotic crossovers are associated with open chromatin and enriched with *Stowaway* transposons in potato

**DOI:** 10.1186/s13059-017-1326-8

**Published:** 2017-10-30

**Authors:** Alexandre P. Marand, Shelley H. Jansky, Hainan Zhao, Courtney P. Leisner, Xiaobiao Zhu, Zixian Zeng, Emily Crisovan, Linsey Newton, Andy J. Hamernik, Richard E. Veilleux, C. Robin Buell, Jiming Jiang

**Affiliations:** 1 Department of Horticulture, University of Wisconsin-Madison, Madison, Wisconsin 53706 USA; 2 USDA-ARS, Vegetable Crops Research Unit, Madison, Wisconsin 53706 USA; 3 Department of Plant Biology, Michigan State University, East Lansing, Michigan 48824 USA; 4 Department of Horticulture, Virginia Tech, Blacksburg, VA 24061 USA; 5 Current address: Departments of Plant Biology and Horticulture, Michigan State University, East Lansing, Michigan 48824 USA

## Abstract

**Background:**

Meiotic recombination is the foundation for genetic variation in natural and artificial populations of eukaryotes. Although genetic maps have been developed for numerous plant species since the late 1980s, few of these maps have provided the necessary resolution needed to investigate the genomic and epigenomic features underlying meiotic crossovers.

**Results:**

Using a whole genome sequencing-based approach, we developed two high-density reference-based haplotype maps using diploid potato clones as parents. The vast majority (81%) of meiotic crossovers were mapped to less than 5 kb. The fine-scale accuracy of crossover detection was validated by Sanger sequencing for a subset of ten crossover events. We demonstrate that crossovers reside in genomic regions of “open chromatin”, which were identified based on hypersensitivity to DNase I digestion and association with H3K4me3-modified nucleosomes. The genomic regions spanning crossovers were significantly enriched with the *Stowaway* family of miniature inverted-repeat transposable elements (MITEs). The occupancy of *Stowaway* elements in gene promoters is concomitant with an increase in recombination rate. A generalized linear model identified the presence of *Stowaway* elements as the third most important genomic or chromatin feature behind genes and open chromatin for predicting crossover formation over 10-kb windows.

**Conclusions:**

Collectively, our results suggest that meiotic crossovers in potato are largely determined by the local chromatin status, marked by accessible chromatin, H3K4me3-modified nucleosomes, and the presence of *Stowaway* transposons.

**Electronic supplementary material:**

The online version of this article (doi:10.1186/s13059-017-1326-8) contains supplementary material, which is available to authorized users.

## Background

Meiosis is a precisely coordinated process where homologous chromosomes undergo pairing and reciprocal exchange of genetic material, ultimately leading to genetically unique haploid gametes. The formation of double strand breaks (DSBs) during the beginning of prophase I marks the initiation of meiosis. Meiotic DSBs are resolved as either crossover (CO) or non-crossover (NCO) events, with the later occurring at a higher frequency [1]. NCOs typically result in the original parental configuration through the synthesis-dependent strand-annealing pathway [2]. COs on the other hand result in the reciprocal exchange of large chromosomal segments between non-sister chromatids and thus contribute to the formation of unique haplotypes and overall genetic diversity for populations of sexually reproducing organisms. COs are also essential for proper chromosomal segregation by providing physical linkages between homologous chromosomes via chiasmata [3]. In most eukaryotes, COs tend to occur in short, 1–2-kb regions, termed crossover hotspots, where crossover rates can be several magnitudes greater than in the surrounding regions [4–6]. Interestingly, the distribution and strength of crossover hotspots display substantial variation among genera, within species, and between sexes [7–11].

The majority of our knowledge of recombination originates from experiments conducted in a few model organisms, including yeast, *Drosophila melanogaster*, and humans [12–14]. However, several key findings have revealed marked differences in the meiotic process for various organisms. In mammalian species, crossover hotspots are established by the presence of a DNA-binding motif of the PR domain zinc finger protein 9 (PRDM9), which specifically tri-methylates histone H3 on lysine 4 (H3K4me3) and directs nucleosome re-organization at these crossover hotspots during early prophase I [15–17]. In contrast, yeast does not contain a PRDM9 homolog [18, 19]. Hotspots in yeast are associated with H3K4me3, occur in regions of low nucleosome density near gene promoters, and are not sequence-dependent. *Drosophila* also lacks PRDM9 and is anomalous due to the absence of crossover hotspots [20]. Meiosis is sex-specific in *Drosophila* since only female meiosis yields crossovers [21]. The absence of unifying characteristics for crossover hotspots among these species suggests that variation in the determinants of meiotic DSB localization is likely a species-specific phenomenon.

Past studies of recombination in plant genomes relied heavily on detecting crossover events from well-defined pedigrees and more recently from population linkage disequilibrium analysis [22–24]. Coalescent-based estimates of recombination rates from linkage disequilibrium studies are available for *Arabidopsis thaliana*, which allowed for the identification of several thousand crossover hotspots [22]. Additionally, it was shown that hotspots in *Arabidopsis* are controlled by the presence of H2A.Z and H3K4me3 and the absence of DNA methylation in all three contexts (CG, CHG, and CHH, where H stands for any nucleotide except guanine) and preferentially occur in gene promoters [22, 25, 26]. However, coalescent-based approaches infer sex-averaged historical recombination rates from heterogeneous populations and thus cannot reveal information pertaining to crossover events resulting from a single sex-specific meiosis. Pedigree analysis has been used to examine differences in male and female meiosis in animal species [27, 28]. The advent of next-generation sequencing technologies has enabled the discovery of millions (M) of sequence polymorphisms, facilitating gains in crossover map resolution. Here, we report the construction of two high-resolution crossover maps in potato using whole-genome re-sequencing. The fine scale nature of our crossover data sets afforded a unique opportunity to investigate the genomic and chromatin features associated with meiotic crossovers in a plant genome.

## Results

### Haplotype map construction

**Fig. 1:**
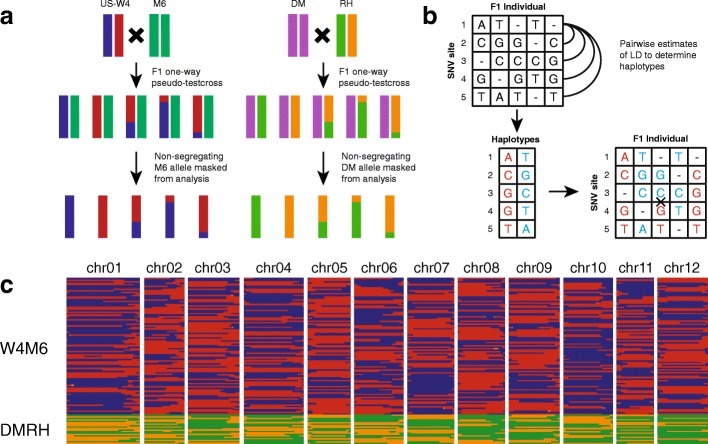
Construction of two high-density haplotype maps. **a** W4M6 and DMRH pseudo one-way testcross populations were used to construct high-density haplotype maps in potato. **b** Using short nucleotide variants (SNVs) to calculate linkage disequilibrium (*LD*) within windows of 50 SNVs, we used high frequency linked alleles to reconstruct the two segregating haplotypes of the heterozygous parent. The reconstructed haplotypes were then used to assign haplotypes to individual SNVs in the progeny. A single crossover between SNV 3 and SNV 4 is illustrated for individual 3. **c** Haplotype map of the W4M6 and DMRH populations. *Red* and *blue* segments reflect the two alleles segregating in the W4M6 population (US-W4 alleles) with *yellow* segments in between to illustrate large crossover intervals (lack of intervening haplotype-differentiating SNVs). The *green* and *orange* segments represent the two alleles segregating in the DMRH population (RH alleles)

A one-way pseudo-testcross population consisting of 90 F_1_ individuals (hereafter termed W4M6) was obtained from an interspecific cross between US-W4 (2*n* = 2*x* = 24), a heterozygous dihaploid *Solanum tuberosum* group Tuberosum clonally maintained genotype derived from a tetraploid potato breeding clone (Minnesota 20-20-34, 2*n* = 4*x* = 48), and M6 (previously referred to as 523-3), a seventh-generation inbred line of the diploid wild species, *Solanum chacoense* (2*n* = 2*x* = 24) (Fig. 1a) [29]. We used a combination of low (1.4–3.5-fold) and high (9–15-fold) coverage whole genome re-sequencing to genotype the W4M6 population and the parents, respectively. Whole genome re-sequencing reads (6.5–13-fold genome coverage) from a second one-way pseudo-testcross population consisting of 20 F_1_ individuals (hereafter referred to as DMRH) from a cross between DM (2*n* = 2*x* = 24), a doubled monoploid *S. tuberosum* group Phureja clone, and RH (2*n* = 2*x* = 24), a heterozygous dihaploid *S. tuberosum* group Tuberosum clone, were obtained from NCBI BioProject PRJNA335820 (Fig. 1a). By mapping reads to the DM v4.04 potato reference genome [30], we identified 1,656,671 and 1,284,653 segregating short nucleotide variants (SNVs; single nucleotide polymorphisms and small, 1–50-bp insertion/deletions) in the W4M6 and DMRH populations, respectively. Thresholds for missing genotype calls were set to 40% for W4M6, and 5% for DMRH per SNV following filtering. A more stringent missing genotype threshold was used for DMRH owing to the smaller population size and higher coverage re-sequencing. Due to the low coverage of re-sequencing in the W4M6 population, segregating SNVs from W4M6 were compared against US-W4 heterozygous genotype calls from next generation sequencing (NGS) and the Infinium 8303 Potato SNP Array [31]. Approximately 99.98 and 95.20% of US-W4 heterozygous genotype calls from NGS reads and the SNP array, respectively, were segregating in the W4M6 population, indicating robust variant identification.

A sliding window approach was implemented to phase SNVs from both populations independently, utilizing estimates of linkage disequilibrium (LD) (Fig. 1b). Alternative alleles were well distributed between the haplotypes of RH and US-W4 (Additional file 1: Figure S1). To overcome sequencing errors, low coverage allele bias, and missing data, we conducted haplotyping using sliding windows of 50 SNV increments and Bayesian inference (Additional file 1: Figure S2). The resulting W4M6 and DMRH maps contained 782 and 155 crossovers, with total map distances of 869 cM and 775 cM, respectively, consistent with previous potato mapping reports that varied from 751 to 965 cM (Fig. 1c) [31, 32].

**Fig. 2:**
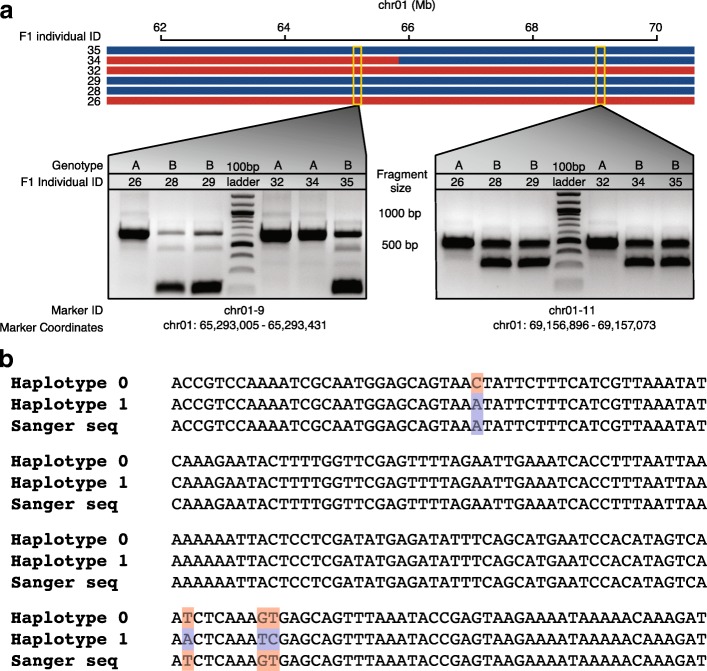
Validation of map construction and crossover resolution. **a** Seven deletion PCR markers were designed using the heterozygous parent US-W4. A random subset of individuals from the F_1_ population (n = 56) was used to screen these markers to validate the SNV-based haplotype map. Here, we illustrate a crossover event between the coordinates of 64 and 66 Mb for F_1_ individual 34 and PCR-based genotyping methodology for a subset of six genotypes. The genotype call is written above the bands for each individual as denoted by the F_1_ individual ID. The only individual with a change in its genotype call (due to a crossover) is the F_1_ individual 34. A single band within a lane represents a homozygote and two bands per lane indicates a heterozygote. Deletion markers were selected based on homozygosity in M6 and heterozygosity in US-W4. *Red*, haplotype 1 and genotype “A”; *blue*, haplotype 2 and genotype “B”. **b** Sanger sequencing of a fine-resolution crossover. The top two sequences are the two US-W4 haplotypes, differentiated by *red* and *blue* polymorphisms, respectively, while the bottom sequence is the result of Sanger sequencing a single crossover from one recombinant individual and is chimeric with respect to the two haplotypes

To validate our haplotype calling of W4M6 on a broad scale, we designed seven PCR primer pairs (Additional file 2: Table S1) surrounding large heterozygous deletions (>50 bp) on chromosome 1 of US-W4, and screened the segregation patterns of the two US-W4 alleles in a random subset of our population (n = 56). Approximately 98% of PCR-based genotype calls were concordant with our SNV-based haplotype map (Fig. 2a; Additional file 2: Table S2).

### Identification and validation of high-resolution crossovers

The high marker density of our data sets enabled identification of crossovers at high-resolution (Additional file 1: Figure S3), yielding median crossover intervals of 880 and 826 bp, for W4M6 and DMRH, respectively (Additional file 1: Figure S4). Crossover counts in non-overlapping 1-Mb windows were significantly correlated between W4M6 and DMRH, considering the DMRH population had substantially fewer crossovers (Spearman’s rank correlation rho = 0.36, *P <* 2.2e^–16^). Subsets of 630 W4M6 and 126 DMRH crossovers were at fine-resolution (less than 5 kb). Of these, 20 and 17 crossovers from the W4M6 and DMRH populations, respectively, had overlapping genomic coordinates. The overlap of W4M6 and DMRH crossovers was significantly greater than by chance (Fisher’s exact test, *P* < 0.026), suggesting that the positions of crossovers are similarly controlled during maternal US-W4 and paternal RH meiosis. Finally, fine-resolution crossovers from both populations were merged and are collectively denoted as the fine-resolution data set (n = 756).

To assess the accuracy of our fine-resolution data set, ten crossovers with a resolution of less than 1 kb were randomly selected for Sanger sequencing (Additional file 2: Table S3). All ten crossovers predicted by whole-genome re-sequencing were confirmed via Sanger sequencing (Fig. 2b). Additionally, greater than 99% (105/106) of SNVs from all Sanger sequenced crossovers were identical to SNV calls from Illumina reads, indicating that our SNV calling procedure is accurate. These results suggest a robust methodology for accurately calling haplotypes and identifying fine-scale crossover in testcross populations.

### Chromosome scale features of meiotic crossovers

**Fig. 3:**
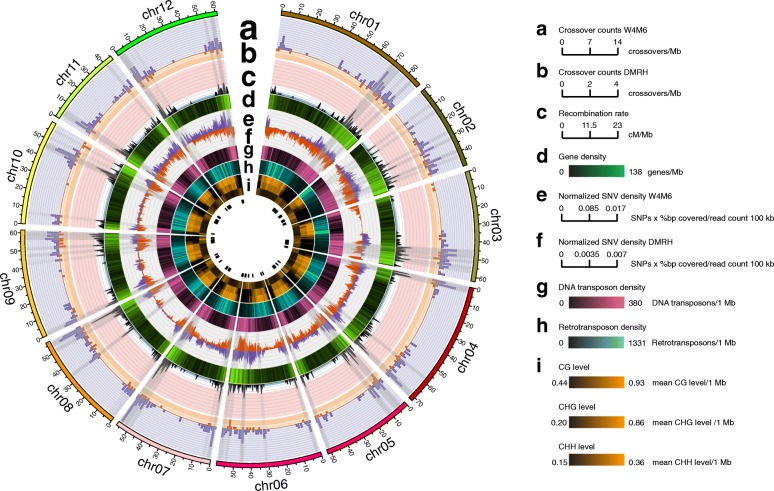
Genomic features of meiotic crossovers. **a** Crossover counts per megabase observed in the W4M6 population (*purple*). **b** Crossover counts per megabase observed in the DMRH population (*orange*). **c** Recombination rate interpolated onto 100-kb windows using crossovers from the W4M6 and DMRH populations collectively. The *red background* of this track denotes the upper third quartile for recombination rate, while the *white background* encompasses 75–25% of recombination rates (second quartile), and *blue highlighted regions* indicate regions below the first quartile of recombination. **d** Gene density per megabase. Normalized SNV density from the W4M6 (**e**) and DMRH (**f**) populations per 100 kb. SNV counts per 100 kb were multiplied by the percentage of bases covered by reads in the 100-kb window and divided by the total number of reads within the window. **g** Count of DNA transposons (class II) per 100 kb. **h** Count of RNA transposons (class I) per 100 kb. **i** Cytosine methylation in CG (first track), CHG (second track), and CHH (third track) contexts expressed as the average methylation level per base, averaged across a 1-Mb window. The *gray windows* indicate regions in the top quartile of recombination rate. The top quartile of recombination rate is also illustrated as *black bars* in the center of the figure

Plotting of all 937 crossovers revealed a preferential distribution towards the distal regions of all chromosomes (Fig. 3a–c), similar to reports in other plants such as tomato and maize [33, 34]. Crossovers were suppressed in the pericentromeric regions of all 12 chromosomes, which are known to be highly heterochromatic and exhibit low gene density (Fig. 3d), high retrotransposon density (Fig. 3h), and high levels of CG and CHG context methylation (Fig. 3i). Furthermore, crossover frequency was positively correlated with gene content (Fig. 3d), SNVs (Fig. 3e, f), DNA transposons (Fig. 3g), and CHH context methylation (Fig. 3i), which highlights the well-known genomic features associated with crossing over in plants [35–37].

**Fig. 4:**
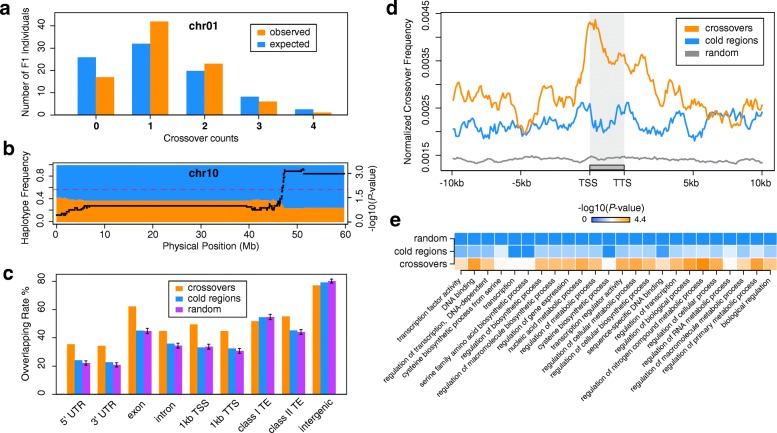
Genomic features associated with meiotic crossovers. **a** An example of the expected versus observed distributions of crossover counts for chromosome 1 of potato. **b** Frequencies of the two W4M6 haplotypes, *blue* and *orange*, overlaid with − log10 transformed *P* values from a chi-squared test for distorted segregation (*black dots*). The *red dashed line* across the plot represents the − log10 transformed significance threshold of *P* < 0.01. **c** Overlap analysis of crossovers with various genomic features (*orange*). Matched “cold regions” were also compared against different genomic features (*blue*), in addition to simulations of random regions (*purple*). Error bars represent the standard deviation of 10,000 simulations. If an interval overlapped more than one genomic feature, the interval was counted towards each overlapping feature. **d** Aggregated plot of crossover frequency relative to all potato genes. Genes were split into 50 windows and up- and downstream regions were split into 25-bp windows. Counts of crossovers were averaged across genes for each window. *Orange*, crossovers; *blue*, cold regions; *gray*, random regions. **e** Heatmap of gene ontology (GO) terms associated with crossover-overlapped, cold, and random genes. No terms were significant in either random or cold data sets, and thus the only GO terms shown are those that were significantly enriched for crossover-associated genes. *P* values are expressed as Benjamini–Hochberg corrected *P* values

We observed substantial variation in average crossover counts across different chromosomes. In *A. thaliana*, average crossovers per chromosome are tightly associated with chromosome length [38]. Similar to *A. thaliana*, the average number of crossovers in potato was positively correlated with chromosome length in base pairs (Pearson correlation; R^2^ = 0.86, *P* < 3.7e^–4^). A Poisson distribution was fit using the average number of crossovers per chromosome to estimate expected crossover counts (Fig. 4a). All chromosomes, except chromosome 3 and chromosome 10, followed the expected distribution, estimated by a multinomial goodness of fit (Additional file 1: Figure S5; Additional file 2: Table S4). Both chromosome 3 and chromosome 10 exhibited higher crossover rates than expected. An overabundance of chromosomes with a single crossover and an underestimate of chromosomes with two crossovers for chromosome 3 are likely a result of insufficient heterozygous SNVs on the short arm of chromosome 3, which effectively masks putative recombination events. Additionally, chromosome 10 was associated with distorted allele segregation in W4M6, which may have played a role in unexpected crossover counts among progeny (Fig. 4b; Additional file 1: Figure S6).

### Fine-scale genomic characteristics of meiotic crossovers

The fine-resolution of our crossover data set (n = 756) provides a unique opportunity to investigate potential associations of crossovers with various genomic features. We explored the overlap between fine-resolution crossovers and 5′ UTRs, 3′ UTRs, exons, introns, promoters (defined as regions 1 kb upstream of transcription start sites (TSSs)), regions 1 kb downstream from transcription termination sites (TTSs), class I and II transposable elements (TEs), and intergenic regions (regions at least 2 kb away from gene TSSs, TTSs, and excluding TEs). We found that crossovers overlapped 5′ UTRs at a rate of 35.45% (268/756) based on the reference genome DM 1-3 516 R44 (DM) annotated gene set [30]. To test whether this overlap is greater than by chance, we performed a Monte Carlo (MC) simulation (10,000×) by permuting a random set of 756 sequences matched by length to the fine-resolution crossover data set from the DM v4.04 reference, and measured the overlap rate of these random sequences with all 5′ UTRs. The mean overlap rate of the permuted regions with 5′ UTRs was 22.19% with a standard deviation of 1.49%, indicating that the overlap rate of the crossover data set with 5′ UTRs is significantly greater than those of random data sets (empirical, *P* < 1e^–4^). Using this methodology for other genomic features, we found that crossovers were significantly associated with 3′ UTRs, exons, introns, promoters, 1 kb regions downstream from TTSs, and DNA transposons (class II TEs), but were negatively associated with RNA transposons (class I TEs) and intergenic regions (Fig. 4c; Additional file 2: Table S5). To make comparisons between crossovers and neighboring regions without crossovers, we constructed a set of 756 genomic locations composed of similar sequence, SNV density, and length as the crossover data set, which were between 10–1000 kb away from a crossover (denoted “cold regions”). In comparison with neighboring “cold regions”, crossovers demonstrated a higher proportion of overlap with 5′ UTRs, 3′ UTRs, exons, introns, promoters, 1-kb downstream regions of TTSs, and a lower proportion of overlap with RNA transposons and intergenic regions, similar to the comparisons with random regions (Fig. 4c; Additional file 2: Table S3). To assess the enrichment of crossovers relative to all genes, we plotted the aggregate crossover density across all genes and their 10-kb surrounding regions (Fig. 4d). Crossovers mainly occurred near TSSs compared to flanking regions, in agreement with the simulation and cold region comparisons.

Next, we investigated the functional annotations of genes or their promoters that overlapped with crossovers. Crossovers were associated with genes related to regulation of biological processes such as “regulation of transcription”, “regulation of a cellular process”, and “transcription factor activity” (Fig. 4e). In contrast, random regions and “cold regions” were not associated with any gene ontology terms (Additional file 2: Table S6). Our results indicate an association between crossovers and genes that play a role in transcriptional regulation.

### Crossovers reside in open chromatin

The enrichment of crossovers near genes prompted us to investigate potential chromatin features associated with crossovers. A genomic region that is hypersensitive to cleavage by DNase I is referred to as a DNase I hypersensitive site (DHS), and is a classic mark of open chromatin [39]. DHSs can be identified by partial DNase I digestion followed by high-throughput sequencing [39]. Another mark suggestive of open chromatin, H3K4me3, was recently shown to be enriched within crossover hotspots in *A. thaliana*, while DNA methylation was notably absent at crossovers [22]. To test for an association between open chromatin features and crossing over, we utilized our recently developed genome-wide DHS data set from DM potato (Zeng ZX, Zhang WL, Marand AP, Buell CR, Jiang JM: Distinct patterns of open chromatin dynamics associated with tissue specifity and response to cold stress, submitted). We used DHSs derived from somatic tissues, as it is not currently feasible to isolate meiocytes for DHS identification in plants. We reason that DHSs consistent across leaf and tuber tissues are likely conserved in most other cell types. A total of 39,205 DHSs was conserved between leaf and tuber tissues (Zeng ZX, Zhang WL, Marand AP, Buell CR, Jiang JM: Distinct patterns of open chromatin dynamics associated with tissue specifity and response to cold stress, submitted). Similarly, we constructed chromatin immunoprecipitation followed by sequencing (ChIP-seq) libraries for H3K4me3 (Zeng ZX, Zhang WL, Marand AP, Buell CR, Jiang JM: Distinct patterns of open chromatin dynamics associated with tissue specifity and response to cold stress, submitted). We identified 14,968 H3K4me3 peaks that were consistent between tuber and leaf tissues.

**Fig. 5:**
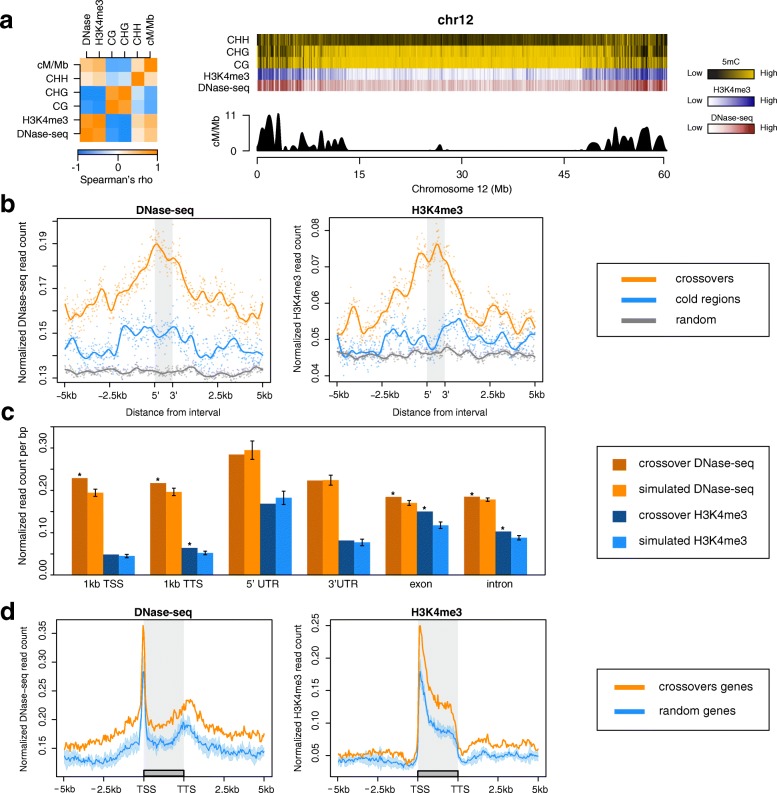
Meiotic crossovers are associated with fine-scale genomic and chromatin features. **a** *Left panel*: Spearman’s correlation matrix for recombination rate, DNase-seq, H3K4me3, and 5mC in CHH, CHG, and CG contexts across 100-kb windows on chromosome 12. *Right panel*: Heatmaps of CHH, CHG, CG, H3K4me3, and DNase-seq density overlaying recombination rate across 100-kb non-overlapping windows of chromosome 12. **b** *Left panel*: Normalized DNase-seq read counts over and flanking crossovers (*orange*), matched cold regions (*blue*), and random regions (*gray*). *Right panel*: Normalized H3K4me3 read counts over and flanking crossovers (*orange*), matched cold regions (*blue*), and random regions (*gray*). **c** Comparison of normalized DNase-seq and H3K4me3 read counts for genomic features overlapping crossovers, and simulations of the analogous features near (10–1000 kb) but not overlapping crossovers. Error bars on the simulated distributions are from 10,000 permutations. **d** *Left panel*: Normalized DNase-seq read counts over and flanking genes and their promoters that overlapped crossovers (*orange*) and permutations of genes that did not overlap genes (*blue*). *Right panel*: Normalized H3K4me3 read counts over and flanking genes and their promoters that overlapped crossovers (*orange*) and permutations of genes that did not overlap genes (*blue*). In both panels the *light blue shading* for non-crossover genes reflects a simulated distribution of 100 permutations, while the statistical tests in the text are based on 10,000 permutations. Genes were split into 50 windows, while flanking regions were split into 10-bp windows. Lines reflect averaged values across genes in each group (crossover-associated and random). *Significance at *P* < 0.05

To determine the chromosome-scale relationships among DHSs, H3K4me3, DNA methylation, and recombination rate, we binned DNase-seq and H3K4me3 ChIP-seq read counts and averaged CG, CHG, and CHH methylation levels in non-overlapping 100-kb windows across the genome. This analysis revealed significant positive correlations for recombination rate with DNase-seq (Spearman’s rank correlation, rho = 0.22–0.43) and H3K4me3 ChIP-seq reads (Spearman’s rank correlation, rho = 0.28–0.59), a weak correlation with CHH levels (Spearman correlation, rho = 0.16–0.36) and strong negative correlation with CG (Spearman’s rank correlation, rho = −0.21 to −0.51) and CHG levels (Spearman’s rank correlation, rho = −0.21 to −0.52) across the 12 potato chromosomes (Fig. 5a; Additional file 1: Figure S7; Additional file 2: Table S7).

Crossover events preferentially occurred within euchromatin, where the distributions of crossovers and open chromatin appear similar on the chromosome level. To address whether crossovers exhibit distinct chromatin architecture on a fine scale, we performed MC simulations (10,000×) comparing the mean DNase-seq and H3K4me3 ChIP-seq read counts overlapping crossovers with randomly permuted nearby cold regions (matched regions 10–1000 kb away, with similar length, gene, GC, and SNV density as crossover regions). Interestingly, crossovers were significantly associated with elevated levels of DNase I hypersensitivity (empirical, *P <* 1e^–4^) and H3K4me3 (empirical, *P <* 1e^–4^) relative to the surrounding euchromatin (Fig. 5b; Additional file 1: Figure S8). Regions of open chromatin are typically regarded as nucleosome-free and are delimited by adjacent nucleosomes. The observation of simultaneously elevated DNase I sensitivity and H3K4me3 in crossovers can be partially explained by taking read count averages across many crossovers coupled with the uncertainty about the precise crossover location within the less than 5-kb intervals. Our data suggest that crossovers preferentially occur in regions of open chromatin and are putatively flanked by neighboring nucleosomes harboring H3K4me3.

DNase I displays a well-known correlation with gene transcription [39]. Similarly, enrichment of H3K4me3 at gene TSSs corresponds to higher gene expression and is frequently associated with active transcription [40]. Of the 756 high-resolution crossovers, 59% (445/756) overlapped a total of 496 genic regions (genes and 1-kb surrounding regions). Crossovers may preferentially occur near or within genes with relatively more accessible chromatin states as a result of active transcription. To test this, we compared the mean DNase-seq and H3K4me3 read counts of crossover-associated genic features (promoters, exons, introns, 5′ UTRs, 3′ UTRs, and 1-kb downstream TTSs) against a similar number of nearby genic features (between 10 and 1000 kb from a crossover) using MC simulations. Interestingly, promoters, 1 kb downstream TTSs, exons, and introns associated with crossovers were significantly enriched with DNase-seq reads compared to simulations (Fig. 5c; Additional file 2: Table S8). H3K4me3 modifications were enriched for regions 1 kb downstream of TTSs, exons, and introns overlapping crossovers, compared to nearby simulated regions (Fig. 5c; Additional file 2: Table S8). Aggregate plots of DNase-seq and H3K4me3 normalized read counts across genic regions associated with crossovers highlight the observation of overall increased chromatin accessibility for genes associated with crossovers (Fig. 5d).

A substantial proportion of crossovers were located near or within genic features. Active genes are generally defined by an overall increase in chromatin accessibility, a feature that may underlie crossover formation. To test whether the association of crossovers with open chromatin is due to genome organization, or an inherent characteristic of crossovers, we surveyed the chromatin state of crossovers specifically residing within intergenic regions. Approximately 77% (583/756) of crossovers overlapped intergenic regions (defined as regions > 2 kb from genes, and excluding TEs). Remarkably, intergenic crossovers were significantly enriched with DNase-seq reads (empirical, *P* < 9e^–3^) and associated with higher levels of H3K4me3 ChIP-seq (empirical, *P* < 1e^–4^) reads compared to permutations of random intergenic regions (within 10–1000 kb), suggesting a preference of crossovers to occur within open chromatin states regardless of genic context (Additional file 1: Figure S9). Furthermore, intergenic crossovers were distinctly associated with elevated levels of open chromatin relative to surrounding regions (Additional file 1: Figure S10). However, while H3K4me3 was statistically elevated in intergenic crossovers compared to intergenic controls 10–1000 kb away, we did not observe a distinct peak over intergenic crossovers relative to directly flanking regions (Additional file 1: Figure S10). This analysis provides evidence that open chromatin is an intrinsic feature of crossovers and the association of crossovers with genic features may be a byproduct of accessible chromatin configurations typically occurring near genes.

### Crossovers are enriched with *Stowaway* DNA transposons

**Fig. 6:**
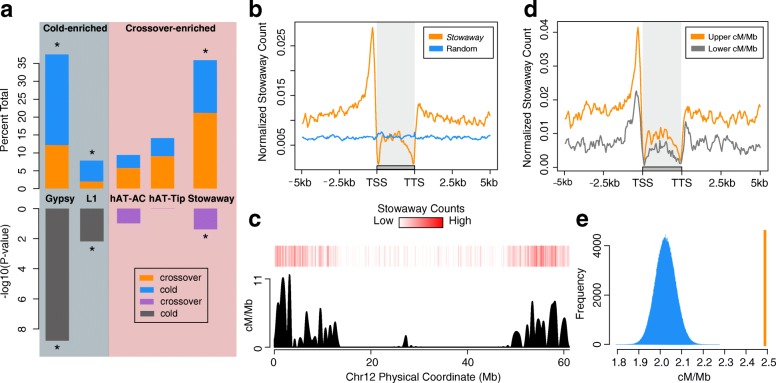
Meiotic crossovers are enriched with *Stowaway* MITE transposons. **a** Significantly enriched (before Bonferroni multiple testing correction) transposons between fine-resolution crossovers and matched cold regions. *Top barplot*: Proportion of significantly enriched transposons in cold regions (*gray background*) and crossovers (*red background*) out of all transposons overlapping each data set. *Blue bars* represent the portion the transposon takes in cold regions. *Orange bars* represent the portion the transposon takes in crossovers. *Bottom barplot*: −log10 transformed *P* values from Fisher’s exact test. *Grey bars* are for transposons enriched within cold regions, *purple bars* denote significance for transposons enriched within crossovers. Transformed *P* values are shown only for the data set in which they are significant. *Significance following Bonferroni correction at *P* < 0.05. **b** Aggregate plot of *Stowaway* element distribution relative to genes across the potato genome. *Orange*, normalized count of *Stowaway* elements (per bp window); *blue*, random control. **c** Heatmap of *Stowaway* elements per 100 kb on chromosome 12, on top of recombination rate interpolated onto 100-kb windows. **d** Promoters of genes from the upper quartile of recombination rate are more enriched with *Stowaway* elements than genes from crossover-poor regions. *Orange*, normalized count of *Stowaway* transposons in gene promoters of recombination rich regions; *gray*, normalized count of *Stowaway* elements within gene promoters from recombination poor regions. **e** Promoters carrying *Stowaway* elements underlie regions with significantly higher recombination rates (*orange line*), compared to simulated distributions (one million permutations) of nearby (within 500 kb) promoters lacking *Stowaway* elements (*blue histogram*). *Y-axis* represents the counts of observations with a mean cM/Mb value (*X-axis*)

Repetitive DNA elements play a prominent role in the establishment of recombination hotspots in humans [41, 42]. Therefore, we evaluated whether there are distinct classes of TEs and repeats enriched within our fine-resolution crossover data set compared with matched recombination cold regions. After applying a Bonferroni multiple test correction, our analysis revealed an association between fine-scale crossovers and a miniature inverted-repeat transposable element (MITE), the *Stowaway* (Fisher’s exact test, *P <* 4.18e^–02^) class of DNA transposons, consistent with our observation of significant overlap of DNA transposons with our crossover data set (Fig. 6a; Additional file 2: Table S9). Cold sequences displayed a strong enrichment of *Gypsy* LTR retrotransposons (Fisher’s exact test, *P <* 1.66e^–9^) and L1 elements (Fisher’s exact test, *P* < 6.62e^–3^), which are classically associated with pericentromeric and recombination-suppressed regions (Fig. 6a; Additional file 2: Table S9). Furthermore, *Stowaway* elements were enriched in W4M6 (Fisher’s exact test, *P* < 6.8e^–3^) and DMRH (Fisher’s exact test, *P* < 3.6e^–2^) crossovers when analyzed as independent populations. Approximately 21% (160/756) of fine-scale crossovers harbored at least one *Stowaway* DNA MITE element, with a total of 206 *Stowaway* elements overlapping all crossovers (average 0.27 *Stowaways* per crossover). MC overlap analysis (10,000×) of crossovers with *Stowaway* elements indicated a strong statistical association using all crossovers (empirical, *P* < 1e^–4^), as well as the W4M6 (empirical, *P* < 2.5e^–2^), and DMRH (empirical, *P* < 3.6e^–2^) populations, autonomously. Additionally, we performed Sanger sequencing on one crossover from each population predicted to contain a *Stowaway* element (Additional file 2: Table S3). Analysis of these two sequences revealed single *Stowaway* elements contained within the validated crossover intervals from both populations (resolution of 365 and 577 bp). These results support the enrichment of the *Stowaway* elements within crossover breakpoints.

We then examined the distribution patterns of *Stowaway* elements genome-wide. A total of 20,247 intact (median length = 224 bp, mean length = 209 bp) *Stowaway* elements was identified using RepeatMasker [43]. *Stowaway* transposons were highly enriched upstream of TSSs and highly depleted within gene bodies (Fig. 6b). Counts of *Stowaway* transposons in non-overlapping 100-kb windows and recombination rate were significantly correlated (Spearman correlation, rho = 0.39, *P <* 2.20e^–16^) (Fig. 6c; Additional file 1: Figure S11). While H3K4me3 (Spearman correlation, rho = 0.43, *P <* 2.20e^–16^) is the most correlated chromatin feature with recombination rate, the correlation between recombination rate and *Stowaway* elements was stronger than recombination rate with DNase-seq (Spearman correlation, rho = 0.34, *P <* 2.20e^–16^), CHH methylation (Spearman correlation, rho = 0.26, *P <* 2.20e^–16^), CHG methylation (Spearman correlation, rho = −0.38, *P <* 2.20e^–16^), and CG methylation (Spearman correlation, rho = −0.38, *P <* 2.20e^–16^) on a genome-wide survey.

To further investigate broad scale associations of chromatin marks and *Stowaway* elements with crossovers, a generalized linear model was fit for crossovers per 100 kb using gene and *Stowaway* counts, DNase-seq reads, H3K4me3 ChIP-seq reads, CG, CHG, and CHH methylation levels in 100-kb windows. Analysis of variance revealed strong significant effects for genes (*P* < 2.2e^–16^) and *Stowaway* elements (*P* < 2.2e^–16^) with weaker, yet still significant, effects from DNase I sensitivity, H3K4me3, CHG, and CHH methylation (Additional file 2: Table S10). Fine-scale analysis using the same variables was also fit using 10-kb windows, revealing that at 10 kb resolution, genes, DNase I sensitivity, and *Stowaway* elements are strong contributors to crossovers, and while still significant, CHH and H3K4me3 had weaker effects on predicting crossover formation (Additional file 2: Table S11).

To determine whether the presence of *Stowaways* in promoters is quantitatively associated with recombination rate, promoters were grouped by either overlapping the top or the bottom quartile for recombination rate at 100 kb resolution. The counts of *Stowaway* elements within promoters from the two groups were compared revealing that *Stowaway* elements are present at a twofold increase within promoters from regions associated with the top quartile of recombination rate compared to regions with low levels of recombination (Wilcoxon rank sum test; *P <* 2.2e^–16^; Fig. 6d). Reciprocally, gene promoters were grouped into two sets, promoters containing at least one *Stowaway* element (n = 3030) and nearby promoters (within 500 kb) lacking *Stowaways* (n = 3030), and compared their overlying 100-kb-scaled recombination rates using MC simulations. Promoters carrying *Stowaways* had significantly elevated recombination rates compared to nearby promoters lacking *Stowaways* (empirical; *P* < 1.0e^–6^; Fig. 6e).

## Discussion

Recent studies in several model organisms have revealed an association between regions with low nucleosome density and meiotic crossovers [17, 22–24, 44]. Historical crossovers in *A. thaliana* are enriched in TSSs containing elevated levels of H3K4me3 and the non-canonical histone variant H2A.Z, while being depleted of canonical nucleosomes and DNA methylation [22]. Crossovers in our populations occurred frequently near gene TSSs, consistent with the genome-wide correlation of recombination rate with genes within 100-kb windows (Spearman’s correlation, rho = 0.39, *P* < 2.2e^–16^). We additionally demonstrate an association between crossovers and regions of DNase I hypersensitivity, consistent with previous findings [23]. DHSs mark regions depleted of bulk nucleosomes [45], which would be favorable to the landing of the recombination machinery during prophase I of meiosis. Crossovers occurred near genes that play a role in transcriptional regulation, possibly implicating specific gene sets that may be actively transcribed during the early stages of meiosis. By partitioning crossovers into groups that overlap genic and intergenic regions, we revealed that chromatin accessibility underlies the establishment of crossovers regardless of the proximity to genes. Additionally, the occurrence of high levels of open chromatin marks is consistent with the presence of *cis*-regulatory elements, which we speculate may play a role in meiotic crossover determination. H3K4me3 was enriched at all crossovers, genic and intergenic, but lacked a distinct signal within intergenic regions relative to flanking sites. This may be due to widespread H3K4me3 marks over larger genomic regions, comprising an overall activating chromatin state that is generally favorable to crossover formation. It is important to consider that the DNase-seq and H3K4me3 ChIP-seq data sets were derived from somatic tissues, and although we only utilize conserved reads between tissue types, our data may not reflect the chromatin architecture of meiotic cells.

Repetitive DNA elements guide recombination events in a sequence-specific manner in humans [41, 42]. In mammalian species, recombination is directed by PRDM9, a C2H2 zinc finger protein which specifically tri-methylates H3K4 during prophase I of meiosis [15, 17]. PRDM9 binds to the CCNCCNTNNCCNC degenerate 13-mer motif, which originated from THE1A/B retrovirus-like retrotransposons, providing some of the first evidence implicating repetitive DNA elements in crossover site determination [15, 16, 42]. We detected a significantly enriched MITE within our crossover data set, an association observed in two independent populations and validated by Sanger sequencing. We demonstrate that the presence of *Stowaway* transposons positively influences recombination rate in gene promoters, although it is critical to note that our estimates of recombination rate are derived from interpolations on 100-kb windows, and thus the low resolution may generalize our results. Genomic regions associated with the top quartile of recombination rate were associated with nearly twice as many *Stowaway* elements per gene promoter compared to promoters underlying regions from the bottom quartile. It is known that *Stowaway* preferentially inserts within gene promoters and forms stable secondary structures [46] and may still be active in potato [47]. The terminal inverted repeats of *Stowaway* elements share an 11-bp consensus sequence, CTCCTCCGTT, which bears a striking similarity to the crossover-associated motifs CTT-repeat and CCN-repeat, identified in *A. thaliana* [22, 24], as well as the human CCNCCNTNNCCNC 13-mer recombination hotspot motif [42]. We found both populations enriched with *Stowaway* elements in crossover intervals. This result may reflect the preference of crossovers and *Stowaway* elements to occur within open chromatin given the preference of *Stowaway* elements to insert in TA-rich sequences depleted of nucleosomes. Furthermore, *Stowaway* transposons leave TA target site duplications, sequence content known to exclude nucleosome binding, leaving chromatin more accessible to meiotic regulatory factors.

A recent experiment revealed that hypomethylated transposons have the potential to contribute functional de novo *cis*-regulatory elements to nearby genes in the form of enhancers, insulators, or repressors [48]. Such events have the possibility to re-wire transcriptional networks in a development and/or environment-specific fashion. Establishment of novel *cis*-elements in transposons is accompanied by increases in regulatory epigenetic marks to the local chromatin. *Stowaway* elements have been previously shown to contain *cis*-regulatory sequences in potato and tomato, including an embryogenesis nuclear factor binding site in carrot [49]. The positional preference of *Stowaway* transposons upstream of TSSs suggests that they may be associated with open chromatin. However, we were unable to assess chromatin accessibility at *Stowaway* transposons due to the short read length (20 bp) of DNase-seq reads, coupled with the high copy and repetitiveness of this TE family. Furthermore, using 20-nucleotide simulated reads derived from the reference genome, we found that less than 46% of *Stowaway* elements had even a single read align, and less than 0.5% of *Stowaway* elements contained the coverage expected. This suggests that our current data set is not suitable for TE analysis, possibly resulting in misleading conclusions. However, with longer ChIP-seq reads (150 nucleotides), we did find an enrichment of H3K4me3 at crossover-associated *Stowaway* elements compared to MC simulations of non-crossover *Stowaway* elements (empirical, *P* < 3.0e^–2^), but the overall levels of H3K4me3 in these groups were substantially lower than random and flanking regions (Additional file 1: Figure S12a). This highlights the well-documented absence of this transcriptional chromatin mark within transposons and may reflect a general depletion of nucleosomes within this TE family. Although we cannot assess chromatin accessibility within *Stowaway* elements, we can, however, examine open chromatin configurations flanking *Stowaway* transposons. Chromatin accessibility was greater for *Stowaway* elements that comprise the top quartile of recombination rate compared to *Stowaway* elements from the bottom quartile (Additional file 1: Figure S12a). Additionally, genes which contain a *Stowaway* element within their promoter have overall elevated chromatin accessibility upstream of TTSs and higher H3K4me3 levels within the gene bodies compared to genes lacking *Stowaway* elements (Additional file 1: Figure S12b). Increased chromatin accessibility for genes associated with *Stowaway* transposons may be more suitable targets for crossovers.

These results raise several questions as to the roles *cis*-regulatory elements and active transcriptional epigenetic marks play prior to DSB formation in meiosis. *Stowaway* elements share a striking number of fine-scale chromatin and genomic features with crossovers, such as preference for nucleosome-depleted regions, AT-rich sequence, and proximity to gene TSSs and H3K4me3-modified nucleosomes. Although *Stowaway* elements are more correlated with recombination rate than several established meiotic chromatin marks, further experimentation will be necessary to establish a mechanistic role for *Stowaway* elements in crossover site determination.

## Conclusions

Meiotic crossovers are largely determined by open chromatin, marked by DNase I sensitivity and the presence of H3K4me3 in the potato genome. *Stowaway* MITEs were also significantly enriched within meiotic crossovers from two autonomous populations, and associated with increased recombination rate. Crossovers and *Stowaway* transposons share a remarkable number of fine-scale chromatin and genomic characteristics. Further investigation into the functional aspects of the chromatin landscape, *Stowaway* elements, and their relationships will be necessary to determine the precise mechanism of crossover site determination in a plant genome.

## Methods

### Plant materials, genomic DNA isolation, and library preparation

A population of 90 F_1_ individuals was developed from an interspecific cross between US-W4 (2*n* = 2*x* = 24), a heterozygous *S. tuberosum* dihaploid derived from a tetraploid Minnesota breeding clone, and M6, a *S. chacoense* clone produced by seven generations of self-pollination. Genomic DNA was isolated from young, emerging leaves of greenhouse grown plants using the DNeasy Plant Mini Kit (Qiagen, Valencia, CA, USA).

Genomic DNA was sheared to 300 bp using a Covaris ultrasonicator. Fragmented DNA was then end repaired, A-tailed, ligated to Illumina compatible adaptors, and PCR amplified for eight cycles. Equal amounts of each library were pooled for gel extraction; the 350–450-bp region was excised from the gel and purified using the QIAquick Gel Extraction Kit (Qiagen). The individual genomic libraries from the F_1_ population were pooled and sequenced in paired-end (PE) mode generating 150-nucleotide reads on the Illumina HiSeq platform. We obtained approximately four to ten million (~6.3 million average) read pairs per individual. Genomic libraries for US-W4 and M6 were prepared as the W4M6 F_1_ population, and sequenced with 100- and 150-nucleotide PE reads, respectively. DMRH raw reads were downloaded from NCBI BioProject number PRJNA335820.

### Read processing and alignment

The quality of the raw Illumina sequencing reads was assessed with FASTQC (http://www.bioinformatics.babraham.ac.uk/projects/fastqc/). Reads were trimmed and filtered using CutAdapt (v1.8.3) [50], requiring minimum base quality of 10 and minimum read length greater than or equal to 75 nucleotides. All reads were aligned to the potato reference sequence genome, DM v4.04 [30], an updated version of the *S. tuberosum* Gp. Phureja DM 1-3 516 R44 whole genome assembly [51]. Reads were aligned using BWA-MEM (v0.7.10) on a per-sample basis using the default parameters [52] and processed with SAMtools (v0.1.19) [53] to extract unique, properly paired reads with mapping quality (MQ) greater than 40. Duplicate reads were marked and removed with PicardTools (v1.119; http://broadinstitute.github.io/picard/). Local realignment of reads around insertion/deletions (indels) was performed using GATK IndelRealigner (v3.4.0) [54].

### SNV calling and filtering

Raw SNVs in both populations were called with FreeBayes (v1.0.2-16-gd466dde), with default parameters [55]. The processing of variants is identical in both populations unless noted otherwise. SNVs overlapping repetitive regions identified by RepeatMasker (v4.0.5; Viridiplantae clade database) were removed [43]. Variants with significant segregation distortion identified by a chi-square test with Benjamini–Hochberg false discovery rate (FDR) set to 0.05 were removed from further analysis. Alleles in a bi-parental population should be highly linked. Therefore, for each variant, we randomly sampled 25 polymorphisms between 1 and 10,000 kb away and estimated linkage disequilibrium in the form of r^2^. Variants with median r^2^ values less than 0.2 were removed. Additionally, since the average coverage for each individual was low (~2× for the W4M6 population), it is likely that only one allele is represented at any given locus. Thus, we set homozygous reference genotypes to missing if the read depth was less than 2 and 5, for the W4M6 and DMRH populations, respectively. The threshold of missing genotype calls allowed per variant site was set to 40 and 5% in the W4M6 and DMRH populations, respectively, owing to the smaller sample size and higher coverage of the DMRH population. All homozygous alternative allele calls (1/1) were converted to heterozygous genotypes (0/1). The rate of homozygous alternative allele calls to the total number of calls was used as the error rate (E_t_).

### Haplotype phase reconstruction

A sliding window was implemented to phase SNVs based on pairwise patterns of linkage disequilibrium. Windows of 100 SNVs with a 1-SNV shift were used to estimate the correlation of linkage disequilibrium (r^2^) to determine associated alleles. The first pair of markers was used to arbitrarily set the haplotypes, by taking the most likely haplotypes as the allelic pairs with the greatest frequencies. Subsequent comparisons were made using a variant that had already been assigned a haplotype. Therefore, subsequent haplotype assignments rely on identifying the two haplotypes with the greatest frequencies and setting the haplotypes of the new marker alleles to the haplotypes of the linked marker alleles. This allows all variant haplotypes to be aligned within a window. When the window slides, the new window overlaps the previous window by 99 SNVs and thus allows the continuation of the phasing algorithm until the end of the chromosome. This results in haplotype assignments for each individual at all SNV positions.

Due to the low coverage and sequencing errors, SNV haplotypes may not reflect the true underlying haplotype for a particular individual in the population. Therefore, we conducted haplotype calling using a window-based approach similar to a method implemented in rice [56]. Briefly, a 50-SNV sliding window, shifting five SNVs at a time with a minimum threshold of five non-missing haplotype calls per window was required to estimate the posterior probability of both haplotypes for a particular individual. The probability of discovering a discordant marker for a given haplotype window of *n* genotyped markers follows a binomial distribution, given the error rate (*E*_*T*_) estimated by the ratio of homozygous alternative allele genotypes to the total number of genotype calls, and the number of markers belonging to the other haplotype, *k* (Eqs. 1 and 2):

1 $$ P\left(k| hap1\right)=\left(\begin{array}{c}n\\ {}k\end{array}\right)\times {E}_T^k\times {\left(1-{E}_T\right)}^{n-k} $$

2 $$ P\left(k| hap2\right)=\left(\begin{array}{c}n\\ {}k\end{array}\right)\times {E}_T^k\times {\left(1-{E}_T\right)}^{n-k} $$

The posterior probabilities for each situation were then estimated using Bayes theorem, where the prior probability, *P*(*hap1*), of either haplotype is 0.5 (Eqs. 3 and 4):

3 $$ P\left( hap1|k\right)=\frac{P\left(k| hap1\right)P(hap1)}{P(k)} $$

4 $$ P\left( hap2|k\right)=\frac{P\left(k| hap2\right)P(hap2)}{P(k)} $$

Haplotypes were called based on the highest posterior probability within a given window (Eq. 5).

5 $$ {P}_{max}(k)=\mathit{\max}\left\{P\left( hap1|k\right),P\left( hap2|k\right)\right\} $$

Adjacent windows with identical segregation patterns were merged, leaving uniquely segregating haplotype skeleton bins. More details on the algorithm, as well as the source code, can be found at https://github.com/plantformatics/phaseLD.

### Determination of crossover breakpoints and recombination rate

Putative crossovers were identified by selecting the haplotype skeleton bins flanking a crossover (adjacent windows of different haplotype assignments). Due to chromosome rearrangements and/or misplaced scaffolds, particularly on chromosome 1, we ordered bins by their genetic positions using MSTMap [57]. Since we are only concerned with crossovers on a fine-scale, we ignored putative crossovers that resulted from flanking haplotype bins that did not physically overlap given their coordinates on the potato pseudomolecules. To determine the precise crossover interval between two SNVs, SNV haplotype calls contained within overlapping adjacent haplotype bins were extracted from the recombinant individual, with SNVs containing missing haplotypes excluded from analysis. Logistic regression was implemented to assign crossover probabilities to each SNV genomic coordinate (independent variable) scored as a binary haplotype (dependent variant, 0 or 1) (Additional file 1: Figure S1). Since we can only define crossovers as occurring between adjacent markers, we estimate the probability of a crossover more precisely as the absolute difference in probabilities assigned to adjacent SNVs. Starting from the SNV pair with the largest crossover probability, we extend outwards to flanking SNVs until the probability of a crossover is greater than 0.95. The extension is accomplished one SNV at a time, always selecting the SNV that improves the crossover probability. We take the crossover intervals as the pair of SNVs that have a probability of a crossover greater than 0.95.

A traditional genetic map was constructed using each 50-SNV haplotype window as a genetic marker, and estimating genetic linkage using MSTMap [57]. The centers of each window were used to create a Marey map with the respective position in centimorgans. Recombination rate was then interpolated onto 100-kb windows using the cubic spline function from the R package MareyMap [58].

### Map validation by Sanger sequencing and PCR analysis

PCR primers were designed to surround ten crossovers identified collectively from the W4M6 and DMRH populations (Additional file 2: Table S3). PCR was performed for 36 cycles of heat denaturation at 95 °C for 30 s, annealing at 55 °C for 30 s, and extension at 72 °C for 30 s after an initial heat denaturation at 95 °C for 3 min. The PCR mix (20 μl) consisted of 1× Ex Taq Buffer (Mg^2+^ plus), 0.2 mM dNTP mixture, 0.5 μM primers, 1 U of TaKaRa Ex Taq polymerase (Clontech, Mountain View, CA, USA), and 250 ng of genomic DNA. PCR products were visualized using gel electrophoresis to confirm the presence of the target band. PCR products were then cloned into the pCR4 plasmid using TOPO TA cloning (Invitrogen). Plasmids with the correct insert size identified using M13 primers (Invitrogen) were subjected to BigDye (Thermo Fisher Scientific) sequencing reactions consisting of an initial 95 °C for 1 min, followed by 44 cycles of heat denaturation at 95 °C for 10 s and 58 °C for 4 min. Reaction clean-up, capillary gel electrophoresis, and laser detection were performed by the Biotechnology Center at the University of Wisconsin-Madison. The predicted haplotype calls and associated nucleotides based on Illumina reads were compared to the aligned Sanger sequencing product.

Heterozygous deletions on chromosome 1 of US-W4 were identified using the software DELLY [59]. PCR primers were designed surrounding 13 heterozygous deletions (Additional file 2: Table S1). Genomic DNA was collected from a subset of individuals (n = 56) of the F_1_ population and from the two parents (US-W4 and M6) using the DNeasy Plant Mini Kit (Qiagen) following the manufacturer's instructions. We initially screened these markers in the parents, and found that ~ 54% (7/13) of these deletions were heterozygous in US-W4 and homozygous in M6. DNA samples were amplified by PCR using these seven primers (Additional file 2: Table S2). PCR was performed for 36 cycles of heat denaturation at 95 °C for 30 s, annealing at 55 °C for 30 s, and extension at 72 °C for 30 s after an initial heat denaturation at 95 °C for 3 min. The PCR mix (20 μl) consisted of 1× Ex Taq Buffer (Mg^2+^ plus), 0.2 mM dNTP mixture, 0.5 μM primers, 1 U of TaKaRa Ex Taq polymerase (Clontech, Mountain View, California), and 250 ng of genomic DNA.

### Assessing the association of crossovers with genomic features

We used a 1-bp minimum overlap between crossovers and various genomic features, and measured the overlapping rate of crossovers with 5′ UTRs, 3′ UTRs, exons, introns, promoters, 1 kb downstream genes, class I and II TEs, and intergenic regions. We then randomly permuted genomic regions using BEDtools shuffle (v2.25.0) [60] and assessed the overlap of these random regions with each feature. This simulation was conducted 10,000 times.

In order to compare the fine resolution crossover data set (n = 756) with a non-crossover control, we developed a set of random, recombination cold regions (n = 756) which satisfied the following criteria: (*i*) similar GC content within 10% of the matched crossover interval; (*ii*) within 10–1000 kb of a crossover; (*iii*) exact same fragment length as the matched crossover interval; (*iv*) similar SNV density (within 10%) to the matched crossover interval; and (*v*) on the same chromosome as the matched crossover interval. The crossover and recombination cold region data sets were assessed for their overlap with 5′ UTRs, 3′ UTRs, exons, introns, 1 kb upstream of TSSs, 1 kb downstream of TTSs, class I and II TEs, and intergenic regions. If a fragment overlapped more than one feature, it was counted toward all overlapped features. Evaluation of these intersections was performed using BEDtools intersect [60]. TEs were annotated using RepeatMasker (v4.05) [43] and the Viridiplantae clade repeat annotation from the RepBase database (http://www.girinst.org).

### Gene ontology enrichment

Genes or their promoters (n = 937) that overlapped crossovers were screened for enriched gene ontology terms using agriGO [61]. A total of 937 random genes were used as the randomized control for gene ontology enrichment. Matched nearby cold regions were also screened for gene ontology enrichment of genes or their promoters they overlapped (n = 937), similarly as for the crossover data set. Enrichment analysis was performed using Fisher’s exact test and the Benjamini–Hochberg FDR *P* value normalization. Background terms were set to all annotated potato genes for each enrichment test.

### Chromatin state analysis

DNase-seq and H3K4me3 ChIP-seq reads were aligned to the DM v4.04 potato reference genome using Bowtie [62] with default parameters. Only reads aligning uniquely were retained. DNase-seq and H3K4me3 reads that overlapped between tuber and leaf data sets were kept for further analysis. For genome-wide correlation analysis, read counts in 100-kb non-overlapping bins were summed for DNase-seq and H3K4me3, and averaged for DNA methylation and compared via Spearman’s correlation.

For aggregate plots, epigenetic mark data were averaged in 10-bp non-overlapping bins flanking up to 5 kb either side of each crossover interval. Since each center feature was variable in length, we divided each feature into 50 windows and normalized the epigenetic mark data based on a per nucleotide basis. The final plots represent normalized averages across noted regions for the given epigenetic mark. The shaded regions surrounding random or cold values demarcate two standard deviations, calculated from the empirical distribution of 100 permutations.

Monte Carlo simulations were used to evaluate the statistical association of DNase-seq and H3K4me3 reads over genic features (UTRs, promoter, exons, introns, and 1 kb downstream TTS) that overlapped crossovers. The mean normalized read count for crossover-associated features were compared to distributions of the same features stemming from permuted regions 10–1000 kb away. A similar approach was utilized for comparing the mean read counts of DNase-seq and H3K4me3 reads overlapping all crossovers, gene-associated crossovers, and intergenic crossovers compared to control regions 10–1000 kb away.

## Additional files

Additional file 1: **Figure S1.** The distribution of alternate alleles across the haplotypes of RH and US-W4. **Figure S2.** Window-based haplotyping by Bayesian inference. **Figure S3.** Identification of crossovers by logistic regression. **Figure S4.** Crossover interval length by data set. **Figure S5.** Comparison of expected and observed crossover counts per chromosome. **Figure S6.** Evaluation of segregation distortion in the W4M6 population. **Figure S7.** Comparison of different chromatin data sets with recombination rates. **Figure S8.** Crossovers have higher DNase-seq and H3K4me3 levels than empirical distributions of nearby regions. **Figure S9.** Intergenic crossovers have enriched DNase-seq and H3K4me3 signals. **Figure S10.** DNase-seq and H3K4me3 levels over intergenic crossovers. **Figure S11.** Recombination rate is correlated with *Stowaway* element density. **Figure S12.** H3K4me3 and DNase-seq at *Stowaway* transposons. (XLSX 37 kb)

Additional file 2: **Table S1.** PCR primers designed to detect heterozygous deletions on chromosome 1 in US-W4. **Table S2.** PCR-based genotypes across chromosome 1. **Table S3.** PCR primers for sanger sequencing of crossovers. **Table S4.** Multinomial goodness of fit for crossover counts at each chromosome based on fit to Poisson distribution. **Table S5.** Monte Carlo simulation for the association of crossover breakpoints at various genomic regions. **Table S6.** Gene ontology enrichment for crossovers against all potato genes. **Table S7.** Spearman’s correlation coefficient (rho) for 100 kb windows across all 12 potato chromosomes. **Table S8.** Enrichment of DNase-seq and H3K4me3 reads in crossover-associated genic features relative to nearby simulated regions. **Table S9.** Fisher’s Exact Test between the count of fine-scale crossovers (n=756) and matched cold regions (n=756) containing different repeat classes. **Table S10.** Generalized linear model of crossover counts per 100 kb using genomic and chromatin features. **Table S11.** Generalized linear model of crossover counts per 10 kb using genomic and chromatin feature. (PDF 6763 kb)
