# Supplementary material for: Meiotic crossovers are associated with open chromatin and enriched with Stowaway transposons in potato
Source: Genome Biol. 2017 Oct 30;18:203. doi: 10.1186/s13059-017-1326-8 (PMC5663088; doi:10.1186/s13059-017-1326-8)
Supplement: Supplementary file 1 — Figure S1. The distribution of alternate alleles across the haplotypes of RH and US-W4. Figure S2. Window-based haplotyping by Bayesian inference. Figure S3. Identification of crossovers by logistic regression. Figure S4. Crossover interval length by data set. Figure S5. Comparison of expected and observed crossover counts per chromosome. Figure S6. Evaluation of segregation distortion in the W4M6 population. Figure S7. Comparison of different chromatin data sets with recombination rates. Figure S8. Crossovers have higher DNase-seq and H3K4me3 levels than empirical distributions of nearby regions. Figure S9. Intergenic crossovers have enriched DNase-seq and H3K4me3 signals. Figure S10. DNase-seq and H3K4me3 levels over intergenic crossovers. Figure S11. Recombination rate is correlated with Stowaway element density. Figure S12. H3K4me3 and DNase-seq at Stowaway transposons. (XLSX 37 kb) [file 13059_2017_1326_MOESM1_ESM.pdf]

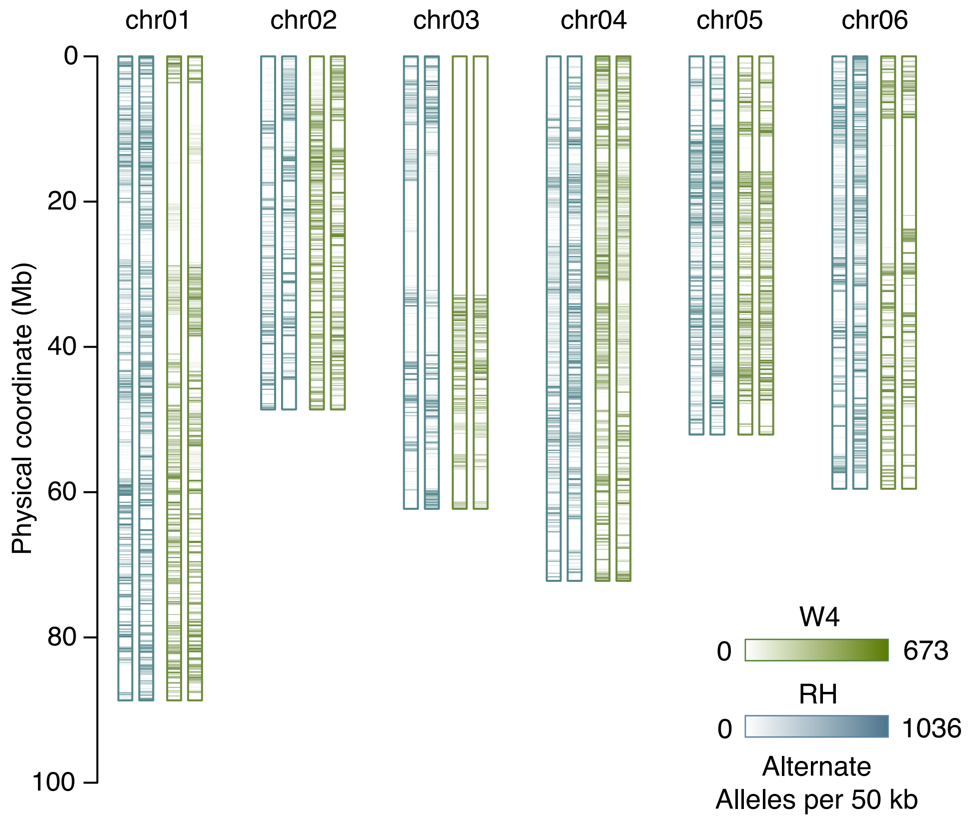

Figure S1 The distribution of alternate alleles across the haplotypes of RH and US-W4. The counts of alternate alleles per 50 kb for each haplotype (for both RH and W4) were summed across all 12 potato chromosomes. The green heatmap reflects the two alleles derived from US-W4 and the density of alternate nucleotides relative to the DM v4.04 reference, while the blue heatmap reflects the two RH haplotypes and the density of alternate nucleotides compared to the DM v4.04 reference sequence.

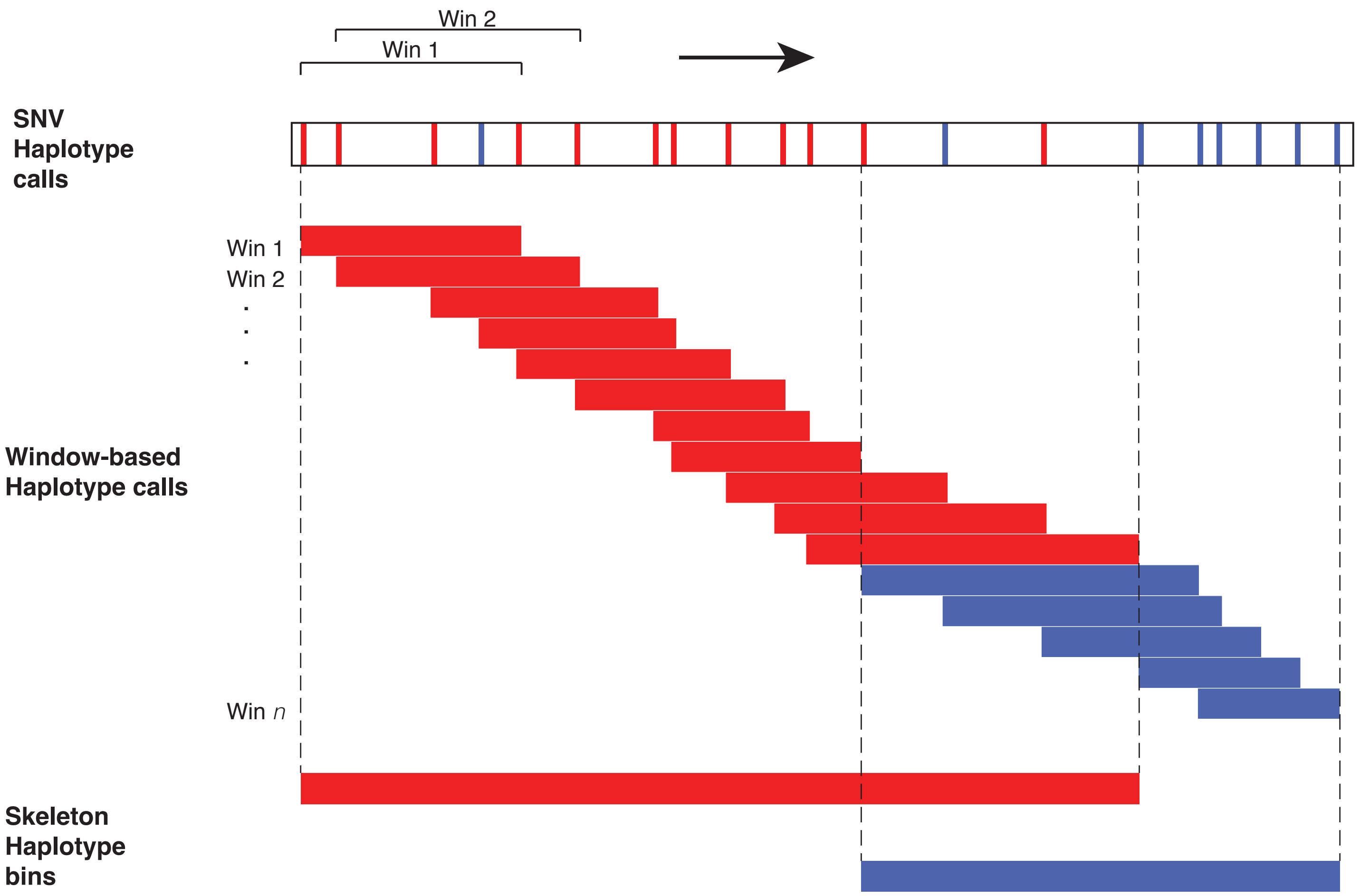

Figure S2 Window-based haplotyping by Bayesian inference. Windows of 50 SNVs, shifting one SNV at a time were used to estimate posterior probabilities for the two segregating haplotypes (1 = red and 2 = blue). Briefly, a binomial distribution was used to estimate the probability a discordant marker given the underlying haplotype and the error rate estimated empirically. The prior probability of each haplotype was set to 0.5 due to the expected segregation ratio of 1:1 for a one-testcross population. Posterior probabilities for each haplotype were calculated using Bayes theorem. For example, in Win 1, there are 4 SNVs with the red haplotype and 1 SNV with the blue haplotype. Using equations 2 and 3 from the Methods section and assuming an error rate of 0.1, P(k I hap1) = 0.33 and P(k I hap2) = 0.00045. Thus, using Bayes theorem and the law of total probability for the denominator, the posterior probabilities for Win 1 are:

$$P(hap1 \mid k) = \frac{0.33 \times 0.5}{(0.33 \times 0.5) + (0.00045 \times 0.5)} = 0.998$$

$$P(hap2 \mid k) = \frac{0.00045 \times 0.5}{(0.33 \times 0.5) + (0.00045 \times 0.5)} = 0.002$$

The haplotype with the highest probability is called for the given window. The algorithm then shifts 1 SNV site and the process continues until the last SNV of the chromosome. Overlapping windows with identical segregation patterns are merged, separating skeleton haplotype bins by a single crossover event, contained within overlapping coordinates.

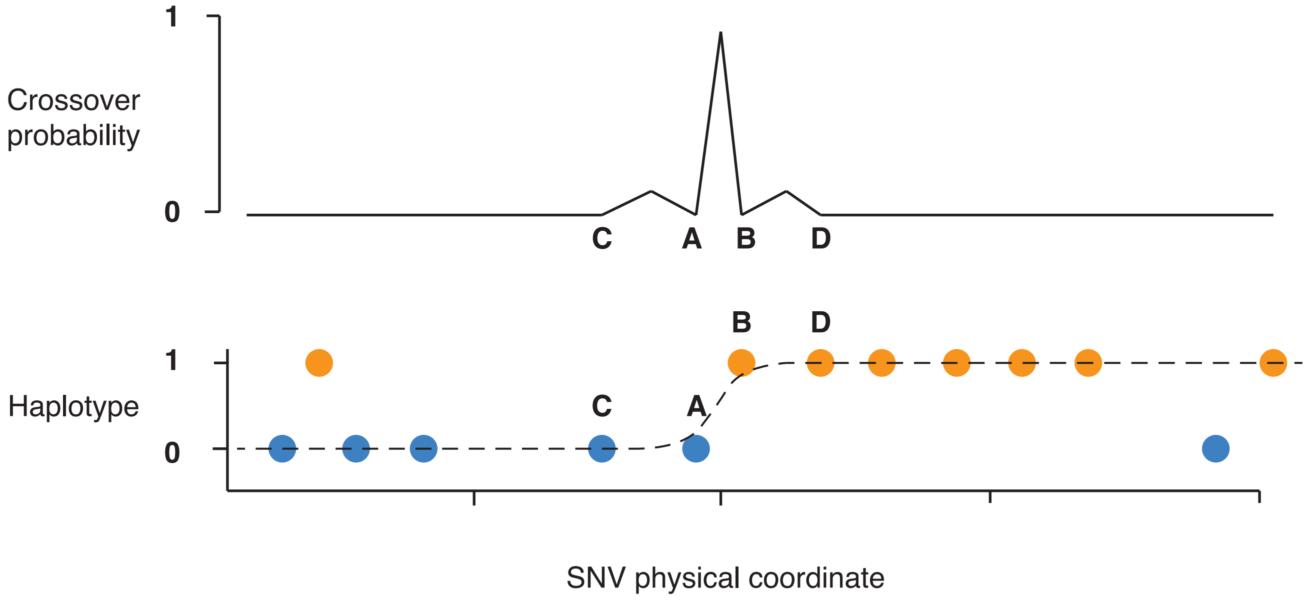

Figure S3 Identification of crossovers by logistic regression. Bottom panel: A logistic regression is fit (black dashed line) to the SNV haplotypes (blue formatted as “0” and orange formatted as “1”). The result is probabilities assigned to each SNV, where the probability of a crossover is equivalent to the probability of haplotype 0 moving to haplotype 1 (black dashed line). Top panel: The interval (flanking SNVs) with the greatest absolute difference in crossover probability (black dashed line), SNVs A and B in this case, are taken as the starting interval. If the difference in probability is greater than 0.95, we take SNVs A and B as the interval containing the crossover. If the difference in crossover probability between A and B is less than 0.95, we extend the interval in either direction from A and B (such as markers C or D, which ever has a greater increases in the probability of a crossover) until the difference in crossover probability is greater than 0.95.

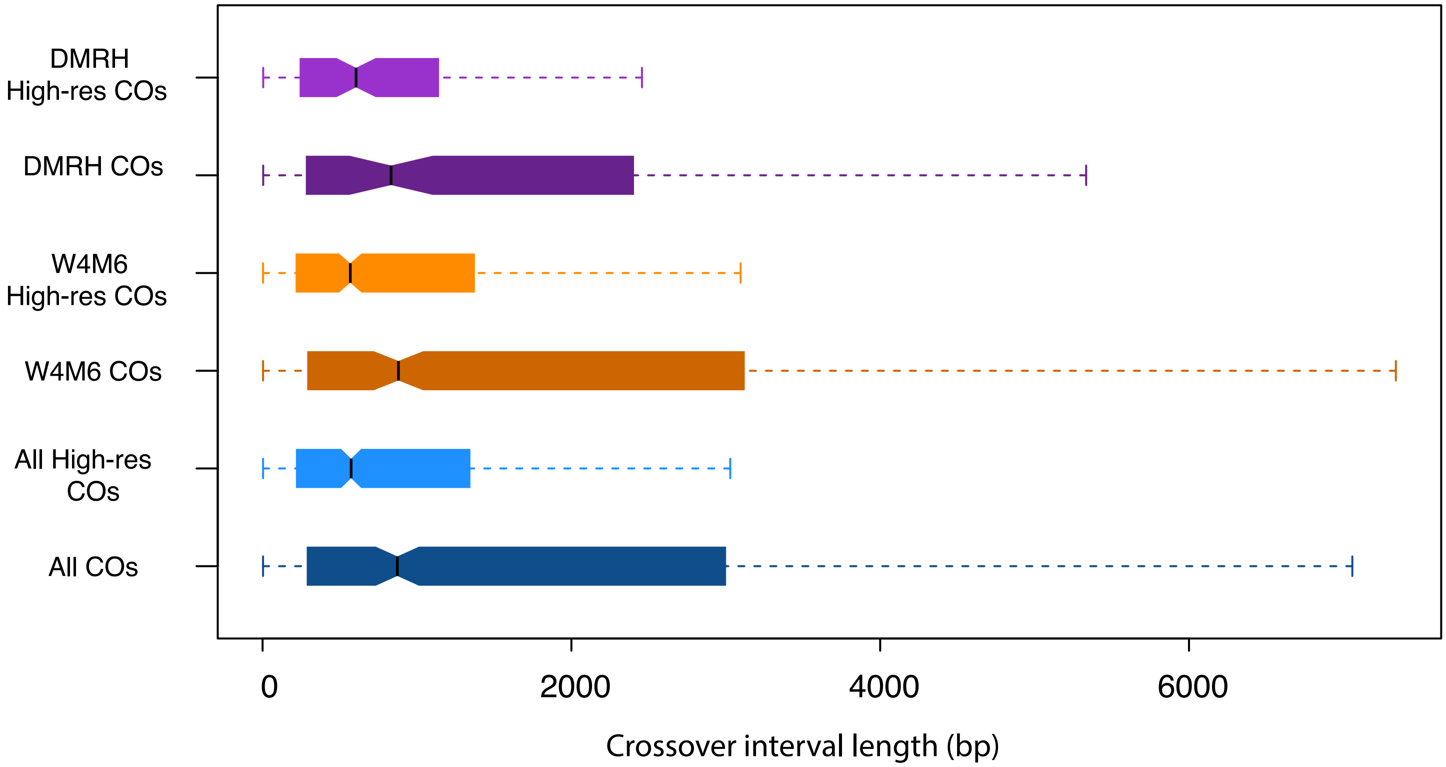

Figure S4 Crossover interval length by data set. The X-axis denotes the length of the crossover interval in base pairs. Y-axis segregates the different crossover data sets. Whiskers extend to 1.5 times the interquartile range. Black bars within each box plot represent the median crossover length.

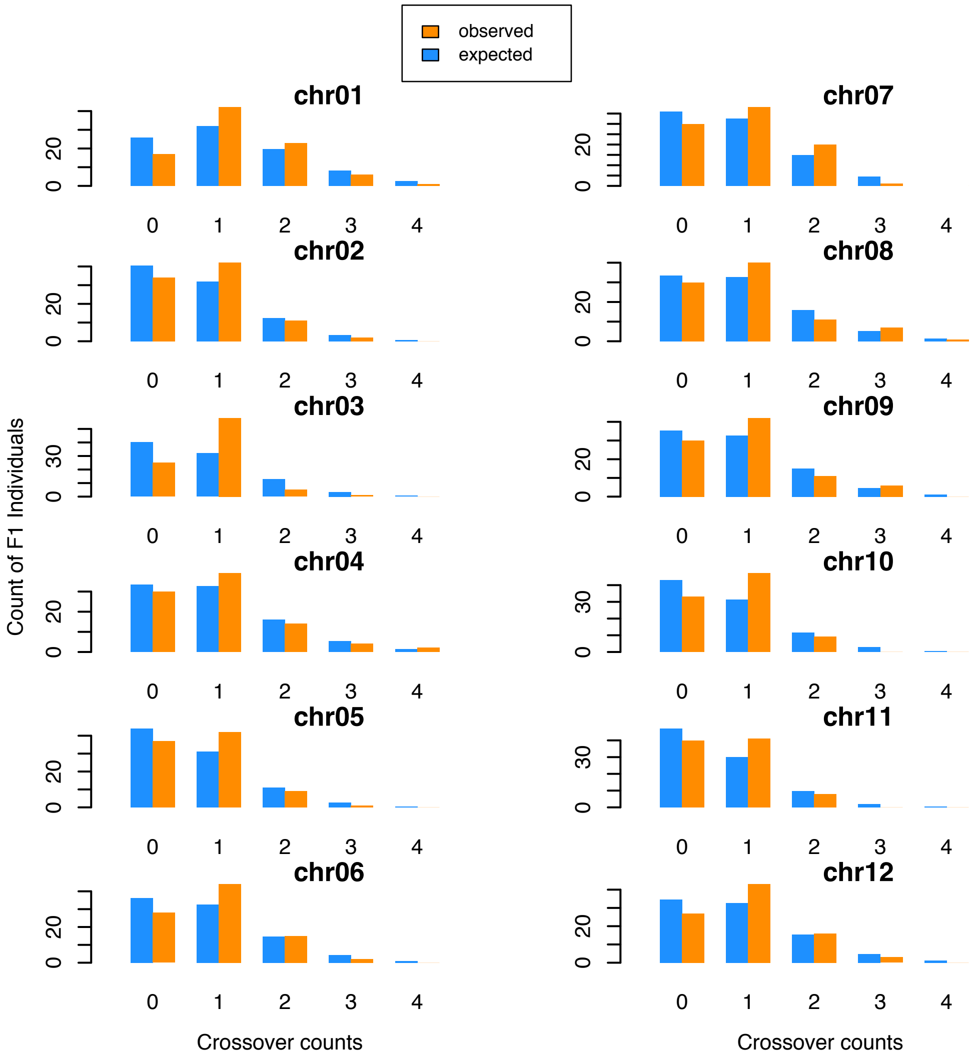

Figure S5 Comparison of expected and observed crossover counts per chromosome. A Poisson distribution was fit using the mean crossover counts per chromosome to estimate expected numbers of crossovers per chromosome. Orange bars are the observed number of chromosomes with a given number of crossovers. The blue bars represent the expected counts of chromosomes with different crossover counts.

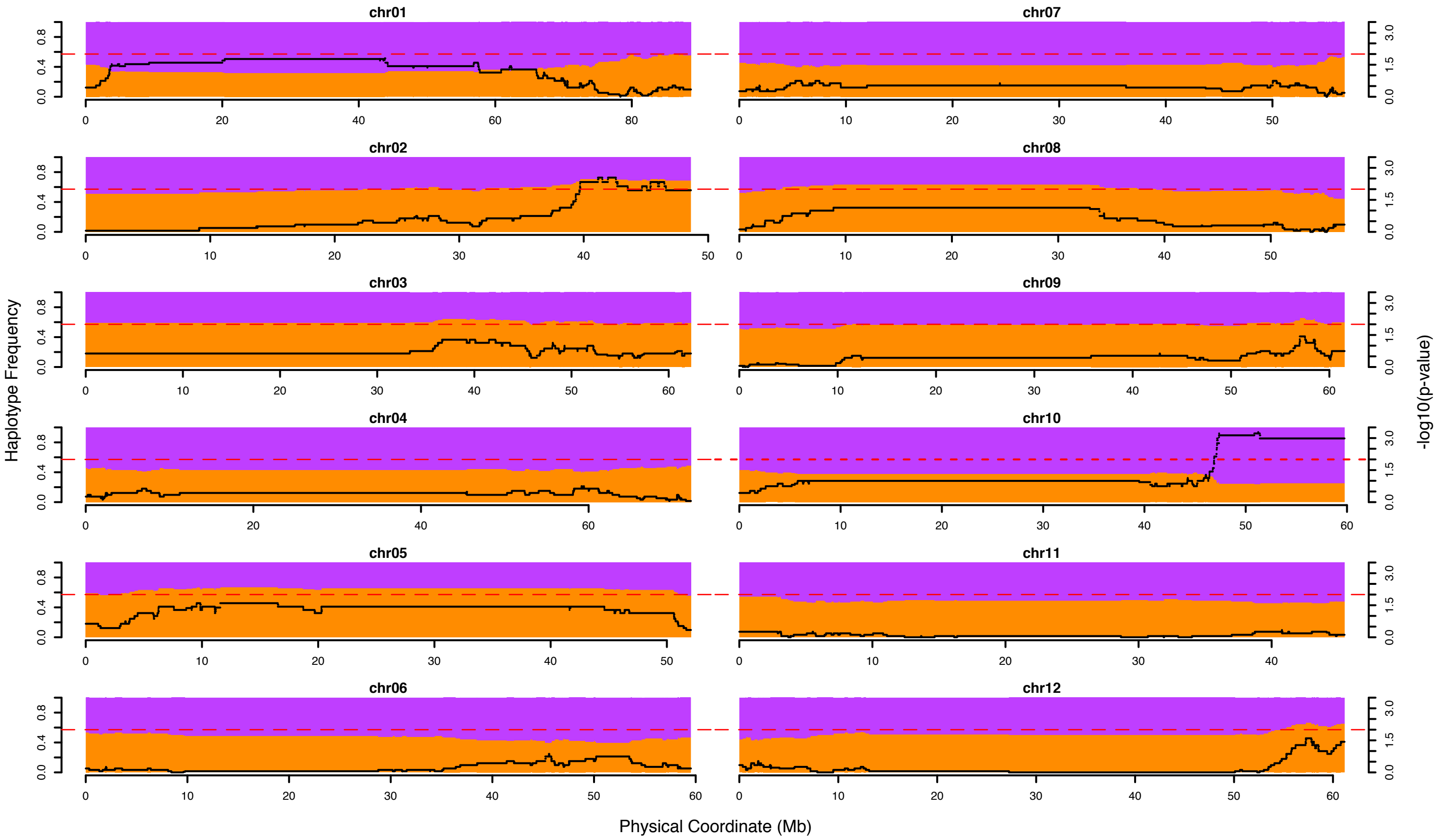

Figure S6 Evalutation of segregation distortion in the W4M6 population.The frequencies of the two haplotypes of W4 are denoted by orange and purple barplots (left Y-axis) and plotted across the physical coordinates of the potato genome (X-axis). A chi-squared test was performed on each bin for deviation from the expected ratio of 1:1. -log10 transformed *P*-values are plotted as black segments (right Y-axis). The red dashed line represents the significance threshold *P*<0.01.

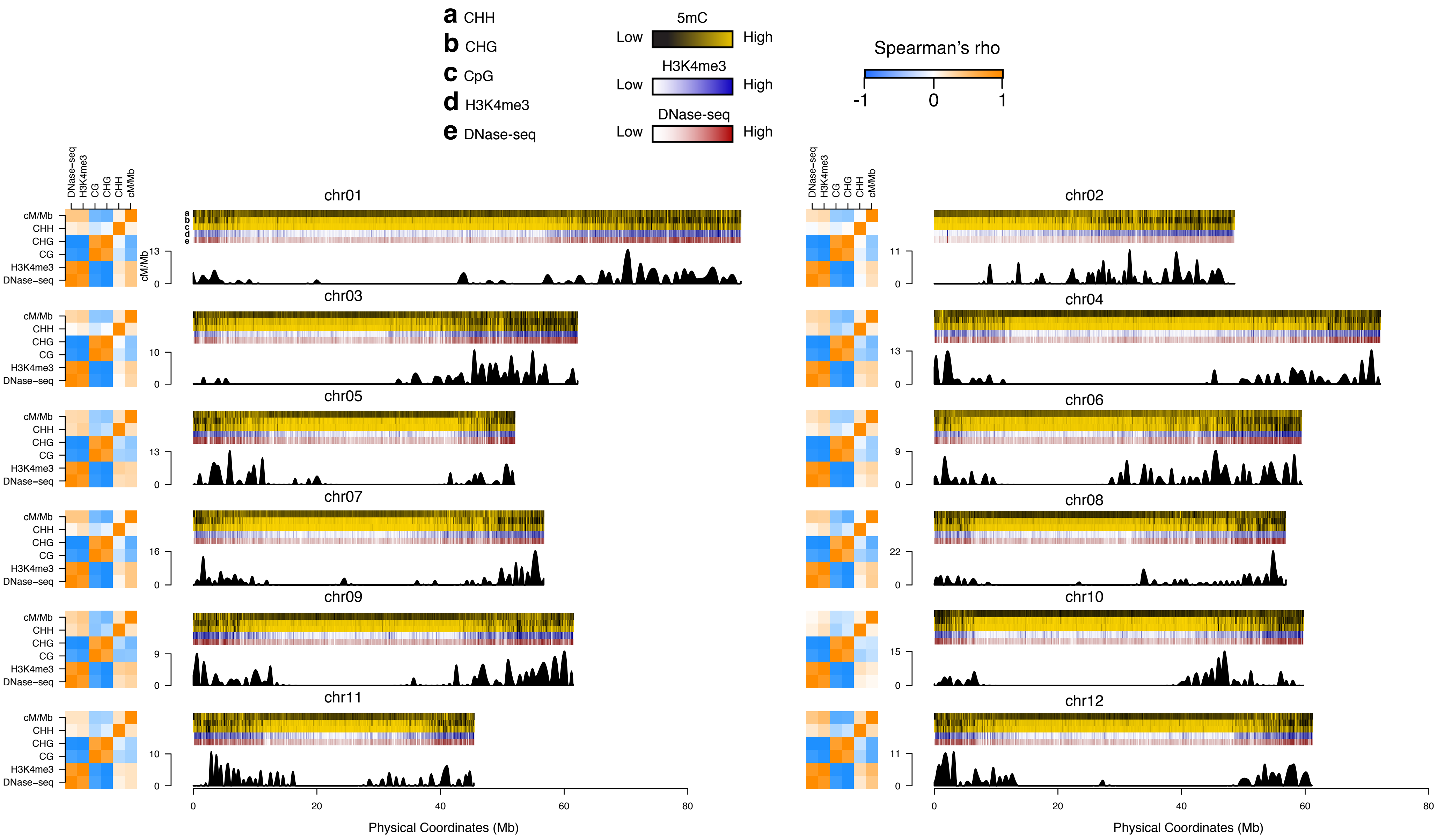

Figure S7 Comparison of different chromatin data sets with recombination rates. Spearman's correlation rho between recombination rate, CHH, CHG, CG, H3K4me3, and DNase-seq using averaged counts (H3K4me3 and DNase-seq) and averaged percentage methylated (5mC) for non-overlapping 100 kb windows. Divergent orange and blue colors represent the level and direction of correlation between data sets. Recombination rate is plotted in black for 100 kb windows across the physical coordinates for each chromosome. Chromatin data sets are plotted above recombination rate as heatmaps in the following order **a** CHH, **b** CHG, **c** CG, **d** H3K4me3, and **e** DNase-seq. DNA methylation is plotted using a color gradient of black (low 5mC) to yellow (high 5mC). H3K4me3 is plotted as white (low H3K4me3) to blue (high H3K4me3). DNase-seq is plotted as white (low DNase-seq levels) to red (high DNase-seq levels).

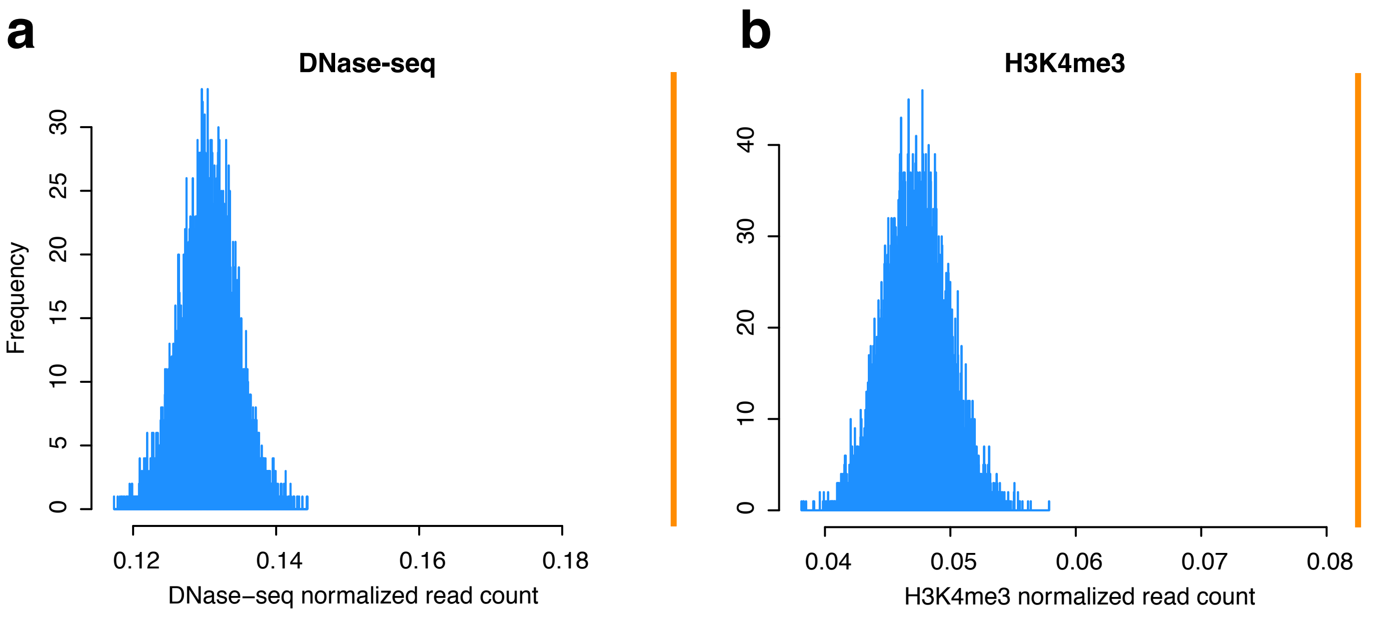

Figure S8 Crossovers have higher DNase-seq and H3K4me3 levels than empirical distributions of nearby regions. Crossover intervals were matched with cold regions within 10-1000 kb and the counts of DNase-seq **a** and H3K4me3 **b** reads were estimated for 10,000 permutations (blue histograms). The orange line is the mean DNase-seq **a** H3K4me3 **b** read counts for crossover intervals.

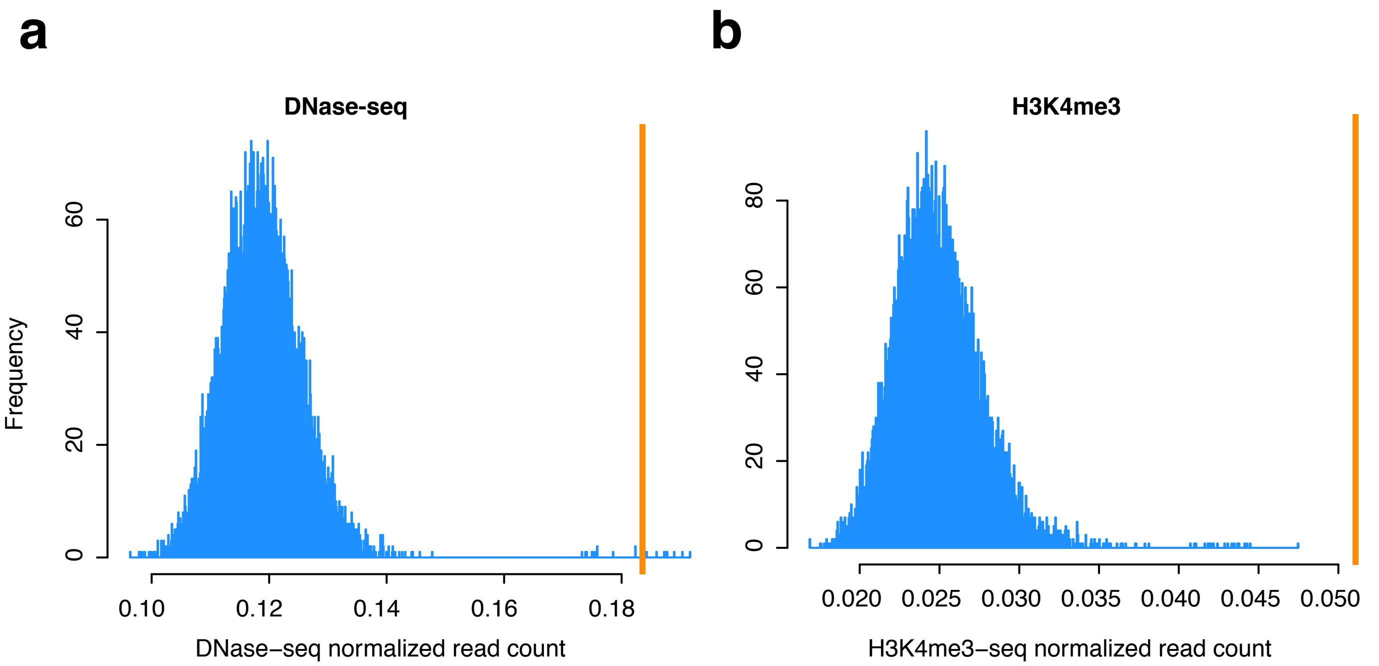

Figure S9 Intergenic crossovers have enriched DNase-seq **a** and H3K4me3 **b** signals. Simulated distributions (10,000x) of matched nearby intergenic cold regions (10 - 1000 kb away from intergenic crossovers) are represented by the blue histograms, where the X-axis indicates the normalized read count and the Y-axis indicates the number of iterations with the cognate normalized mean read depth.

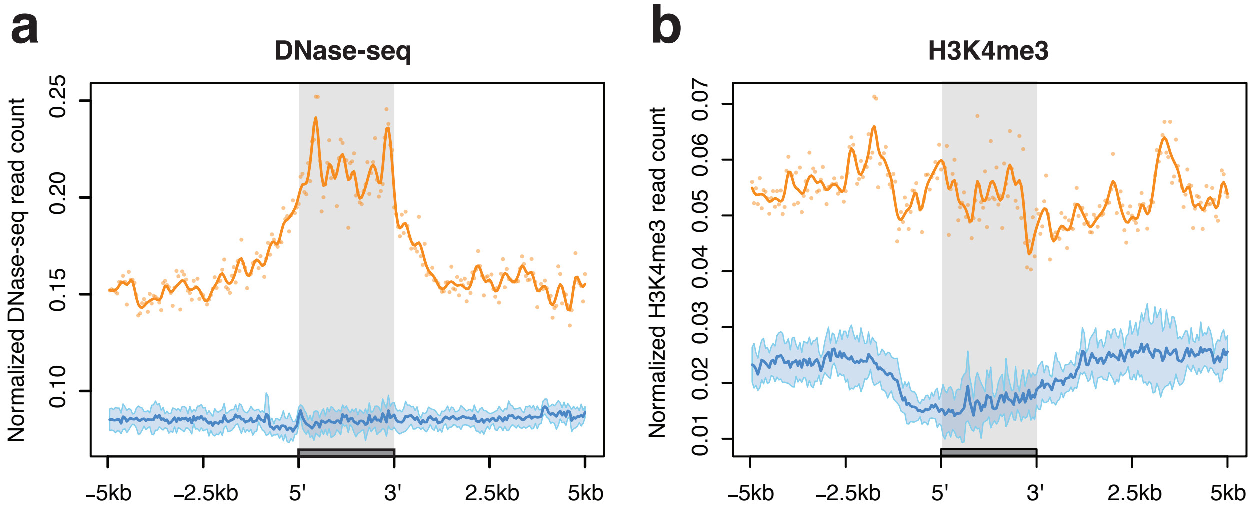

Figure S10 DNase-seq and H3K4me3 levels over intergenic crossovers. Plots represent aggregate values of DNase-seq **a** and H3K4me3 **b** read counts over intergenic crossovers split into 50 windows, and the neighboring 5 kb regions split into 10 bp windows (orange lines). Intergenic crossovers were compared to simulations (100x) of nearby cold regions within 10-1000 kb, blue line. Blue shading represents two standard deviations from the simulated mean.

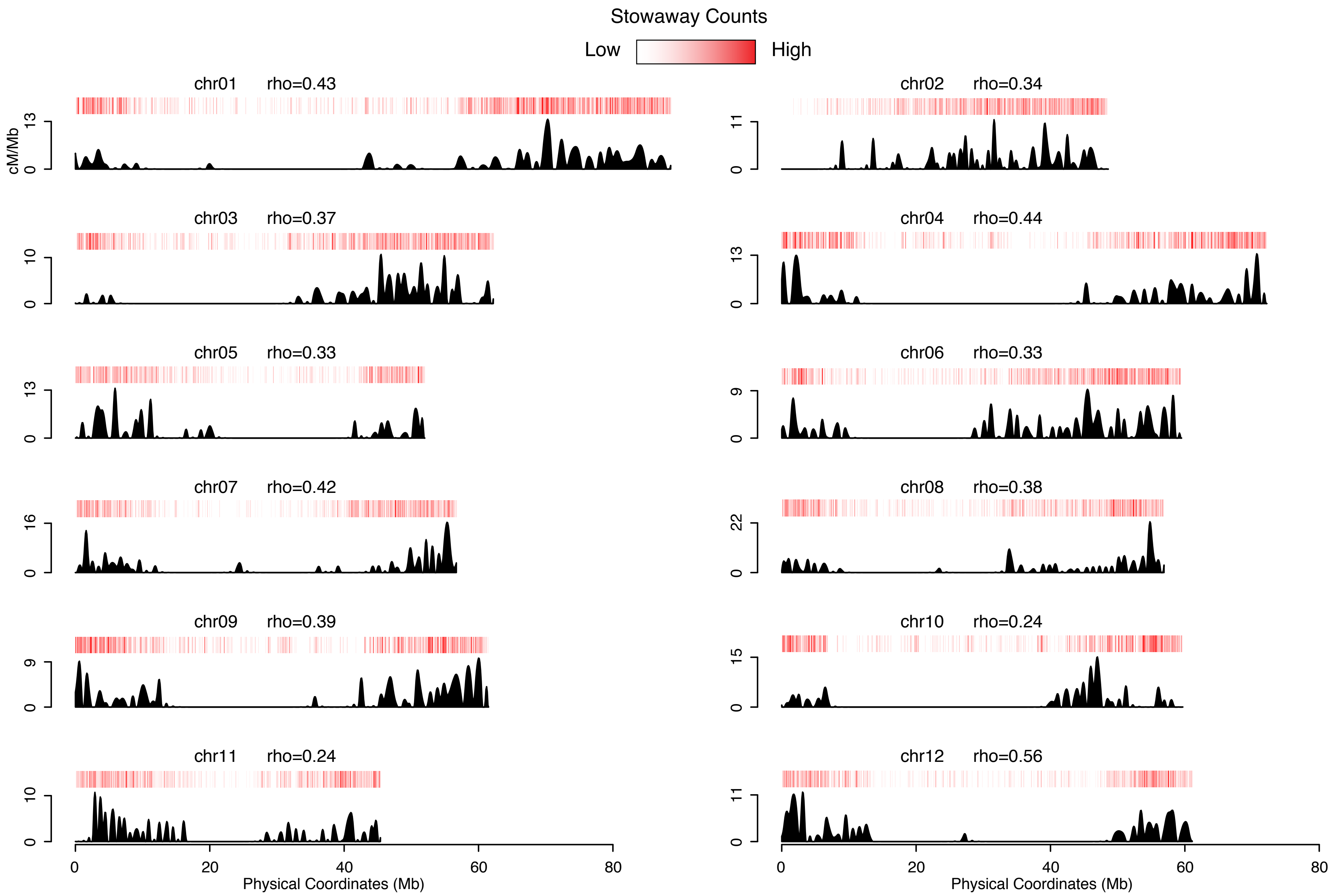

Figure S11 Recombination rate is correlated with Stowaway element density. Recombination rate (cM/Mb) per 100 kb is plotted in black against the physical coordinates of each chromosome. The counts of Stowaway elements are plotted as heatmaps above recombination rate for each chromosome. The divergence of white (low) to red (high) reflects the distribution of Stowaway counts per 100 kb for each chromosome. Spearman's correlation coefficient, rho, for comparisons of recombination rate and Stowaway counts per 100 kb is printed next to each chromosome.

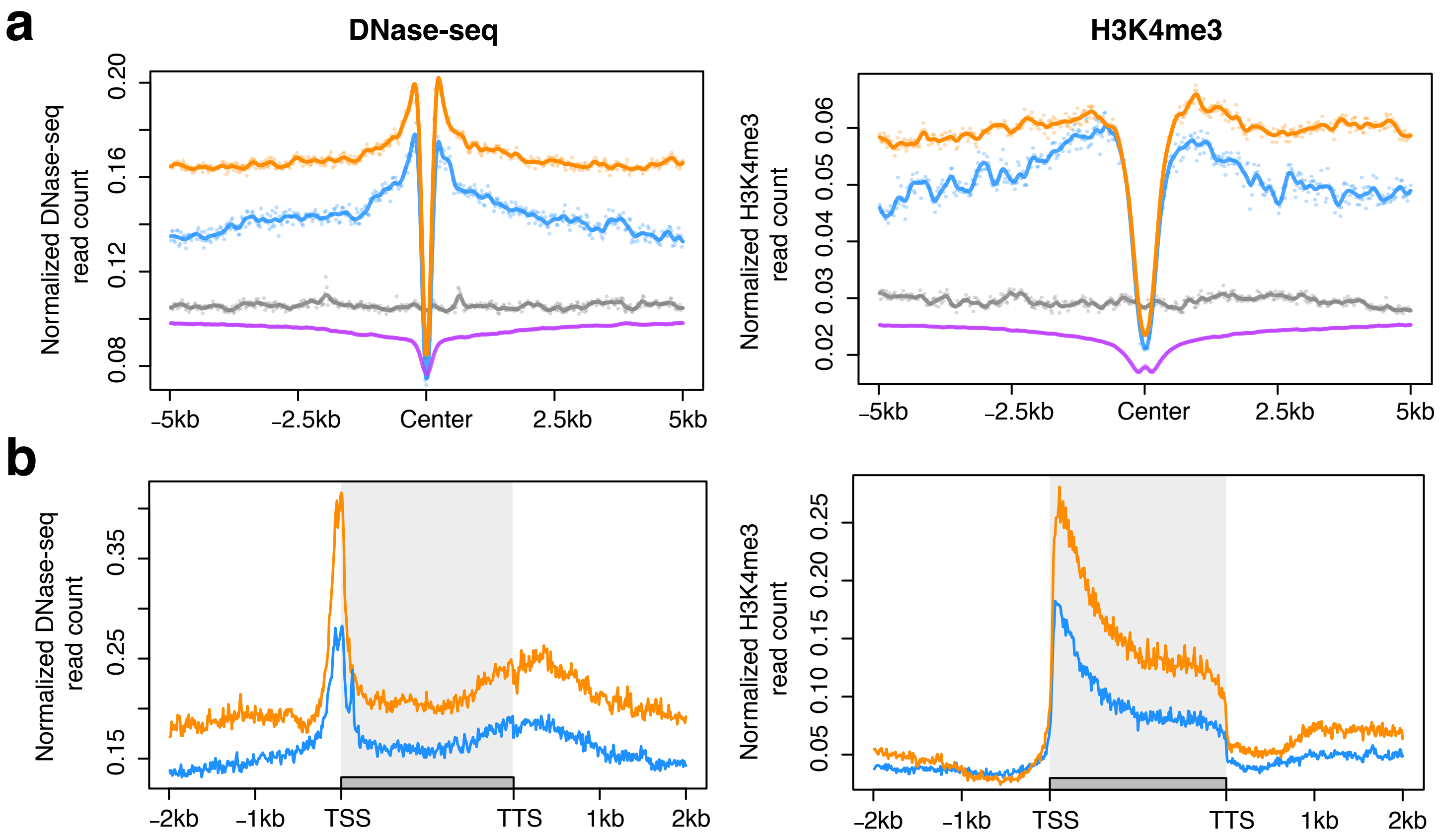

Figure S12 H3K4me3 and DNase-seq at *Stowaway* transposons. (**a**) The levels of normalized DNase-seq (left panel) and H3K4me3 (right panel) read counts were averaged across *Stowaway* elements in the top quartile of recombination rate (orange) and *Stowaway* elements in the bottom quartile of recombination rate (blue). Grey lines represent a random control. The purple lines reflects averaged DNase-seq and H3K4me3 read counts across all transposons. (**b**) Normalized DNase-seq (left panel) and H3K4me3 (right panel) read counts for genes containing *Stowaway* elements in their promoters (orange) and gene promoters lacking *Stowaway* elements (blue).
